# Multi-gene metabolic engineering of tomato plants results in increased fruit yield up to 23%

**DOI:** 10.1038/s41598-020-73709-6

**Published:** 2020-10-14

**Authors:** José G. Vallarino, Szymon Kubiszewski-Jakubiak, Stephanie Ruf, Margit Rößner, Stefan Timm, Hermann Bauwe, Fernando Carrari, Doris Rentsch, Ralph Bock, Lee J. Sweetlove, Alisdair R. Fernie

**Affiliations:** 1 Max Planck Institute of Molecular Plant Physiology, Am Mühlenberg 1, 14476 Potsdam-Golm, Germany; 2 Plant Physiology Department, University of Rostock, Albert-Einstein-Str. 3, 18059 Rostock, Germany; 3 Instituto de Fisiología, Biología Molecular Y Neurociencias (IFIBYNE-UBA-CONICET), Universidad de Buenos Aires, C1428EHA Buenos Aires, Argentina; 4 Institute of Plant Sciences, University of Bern, Altenbergrain 21, 3013 Bern, Switzerland; 5 Department of Plant Sciences, University of Oxford, South Parks Road, Oxford, OX1 3RB UK

**Keywords:** Biotechnology, Plant sciences

## Abstract

The capacity to assimilate carbon and nitrogen, to transport the resultant sugars and amino acids to sink tissues, and to convert the incoming sugars and amino acids into storage compounds in the sink tissues, are key determinants of crop yield. Given that all of these processes have the potential to co-limit growth, multiple genetic interventions in source and sink tissues, plus transport processes may be necessary to reach the full yield potential of a crop. We used biolistic combinatorial co-transformation (up to 20 transgenes) for increasing C and N flows with the purpose of increasing tomato fruit yield. We observed an increased fruit yield of up to 23%. To better explore the reconfiguration of metabolic networks in these transformants, we generated a dataset encompassing physiological parameters, gene expression and metabolite profiling on plants grown under glasshouse or polytunnel conditions. A Sparse Partial Least Squares regression model was able to explain the combination of genes that contributed to increased fruit yield. This combinatorial study of multiple transgenes targeting primary metabolism thus offers opportunities to probe the genetic basis of metabolic and phenotypic variation, providing insight into the difficulties in choosing the correct combination of targets for engineering increased fruit yield.

## Introduction

The yield of the harvested organs of crop plants is influenced by both developmental and metabolic processes^1–4^. While the green revolution was underpinned by the former^5^, major international projects to generate future high yielding crops such as the C4 rice project^6,7^, RIPE^8–10^, project and CASS^11,12^ are increasingly focused on the latter. Indeed, there is ample evidence that the net capacity for assimilation of carbon (C) and nitrogen (N) and their subsequent metabolism into the main cellular biomass polymers is a major determinant of crop yield^13–16^. For example, an analysis of the historical yield gains achieved in wheat demonstrate that recent yield increases were related to increased photosynthesis and enhanced production of stem CHO reserves^17^. Furthermore, transgenic interventions have demonstrated that plant growth and yield can be improved by enhancing the catalytic activity of specific enzymes^18–24^.

Given the strong need for crop yield improvement there is a substantial interest in the engineering of key metabolic processes for increased source-to-sink C and N flows. There are several major challenges in such engineering projects: first it must be decided which are the key metabolic processes; second, an engineering strategy to increase flux of those processes must be designed; and third the necessary genetic changes to implement this strategy must be made. In choosing the key metabolic processes, researchers have tended to focus either on source processes (e.g. the metabolic assimilation of inorganic C into organic precursors^25–30^); or on sink processes (e.g., the synthesis of starch, lipid or protein in tubers, fruits or seeds^31–35^). This choice is usually a pragmatic one: there is a limit to the number of genetic interventions that can be made and therefore it makes sense to focus on the process that is thought to impose the greatest limitation on the overall source-to-sink flow. Essentially, this reduces to an argument as to whether a particular crop is source- or sink-limited. Many of the recent consortium projects to increase crop yield are predicated on the argument that crops are source limited^36,37^, and are thus focusing on source processes such as photosynthesis and N assimilation.

Considerable experimental data is in support of theoretical assessments that both source and sink metabolisms co-limit whole plant fluxes. That said, modulation of net C flow by simultaneous modification of source and sink processes^38,39^, or alternatively genetically modification of C fluxes via manipulation of individual processes of either source or sink tissues such as photosynthesis^40,41^ or carbohydrate synthesis^31–33,42–44^, respectively, have led to increases in plant growth and yield^30,45^. Moreover, Nunes-Nesi et al.^2^ showed that regulation of source-sink interactions is also depending on developmental stage and environmental conditions. Most importantly, there is a strong argument to be made that simultaneous manipulation of source and sink processes lead to a considerable yield increases^39,46,47^. This is mainly due to signals that communicate and regulate the mechanisms of shifting C flow between source and sink tissues. The potential of this strategy is demonstrated by the only experiments to date to make targeted manipulations of both source and sink^39,48^. First, expression of transgenes in potato leaves to increase the partitioning of photoassimilates towards sucrose and away from starch was combined with over expression of two transporters to increase the capacity for starch storage in the tuber^39^. This led to an impressive doubling of potato tuber yield and starch content per plant. Secondly, these studies were achieved with minimal genetic intervention (combined expression of three and one gene – albeit in two specific cell types, respectively). However, the same argument about redistribution of metabolic control applies equally to the local metabolic network as it does to source and sink. For example, it has been suggested that the failure of overexpression of glutamine synthase to consistently increase N assimilation in transgenic crops is due to the lack of simultaneous manipulation of downstream enzymes and transporters^49,50^. The aim of the current study was therefore to use genetic engineering to relieve potential flux bottlenecks at multiple points in the metabolic networks of both tomato leaves, phloem and fruits with the purpose of substantially increasing fruit yield. To do so we took the emergent combinatorial biolistic transformation approach which promises to revolutionize plant metabolic engineering^51^. This approach relies on two unique features of biolistic transformation: (1) the regular integration of multiple copies of transgenes, and (2) their usual integration into a single chromosomal locus^51,52^, with in principle no limit to the number of transgenes that can be integrated simultaneously. Indeed, this route has been taken to achieve increases in three vitamins in maize through the simultaneous integration of five transgenes^53^. Although impressive, the pathways targeted were easy to engineer because of their position at the periphery of the metabolic network and because of known enzyme deficiencies in each of these pathways in maize^54^. We aimed to considerably advance the state-of-the-art by systematically manipulating the core of the metabolic network, a substantially greater challenge because of the larger number of targets that we envisage (up to 20 transgenes) and the distributed control of flux in central metabolism. We assessed the transgenic plants that we created with regard to the expression levels of the introduced genes, their photosynthetic parameters and their metabolite composition. The results are discussed in terms of the overall success of the approach and the implications they have for similar scale metabolic engineering approaches in the future.

## Results

### Generation of tomato plants modifying source and sink metabolisms

Sugar and amino acid accumulation in sink organs is impacted by multiple metabolic and transport processes, ranging from CO_2_ and NO_3_ assimilation to the storage and consumption of the products of these assimilation in sink tissues. We here engineered both source and sink tissues by creating transgenic tomato plants containing up to 20 genes involving in different metabolic and transport processes. These target genes were selected based on the characterization of their effects in single-gene transgenic plants and demonstrated to have positive effects on source or sink carbon or nitrogen flows (Table 1).

**Table 1:** Gene target for enhanced source-to-sink flux in tomato.

Transgene	Full name	Organism from which the genes are derived from	Used promoter	Targeted organism	Type of manipulation	Gene expression		Yield		Rationale	References
						Percentage change	Measured in	Percentage change	Measured in		
*mMDH*	Mitochondrial malate dehydrogenase	Tomato	CaMV35S	Tomato	Knockdown	-45 to -73%	Leaves	10–35%	Fruit (dry weight)		^75^
						-20 to -31%	Fruits				
*SBP*	Sedoheptulose 1,7-bisphosphatase	Arabidopsis thaliana	CaMV35S	Tobacco	Overexpression	150%	Leaves	12%	Biomass		^21^^;^^98,99^
				Tobacco	Overexpression	50%	Leaves	22%	Biomass		
		Tomato		Tomato	Overexpression	30 to 230%	Leaves	4.5–45%	Biomass		
		*Brachypodium distachyon*	Rice tungro virus promoter (RTVP)	Wheat	Overexpression	143 to 176%	Seeds	5–35%	Biomass		
*SPA*	Sugar partitioning affected	Tomato	CaMV35S	Tomato	Knockdown	-80 to -90%	Leaves	11–20%	Fruit (fresh weight)		^68^
						-25 to -82%	Fruits				
*PP*	Pyrophosphatase	*E. Coli*	*cyFBPase*	Potato	Overexpression	145 to 172%	Leaves	56%	Tuber (fresh weight)		^39^
*GS2*	Glutamine synthetase 2	Tobacco	Leaf-specific soybean ribulose-1,5-bisphosphate carbox ylase/oxygenase small subunit gene promotor	Tobacco	Overexpression	15 to 18 (fold change)		20–30%	Biomass		^100^
*GLDH*	H-protein of glycine decarboxylase	*Flaveria pringlei*	*Nuclear photosynthetic gene (ST-LS1)*	Arabidopsis	Overexpression	1.5 to 5	Leaves	37%	Biomass		^24^^;^^79^
		*Arabidopsis thaliana*	CaMV35Sand ST-LS1	Tobacco	Overexpression	5 to 42 (fold change)	Leaves	26–47%	Biomass		
*SWEET 11*	Efflux transporter 11			Arabidopsis	Mutant			− 20 to − 35%	Rosette diameter		^101^
*SUC2/SUC9*	Sucrose transporter 2/9	*Arabidopsis thaliana*	*Cell-specific promoter from Commelina yellow mottle virus (CoYMVp)*	Arabidopsis	Overexpression	2 to 2.5		146%	Companion cells	Enhances phloem loading	^102^^;^^103^
			Plasma membrane H + -ATPase PMA1 gene	Yeast	Overexpression			Increase	Yest cells	Increase uptake capacity of hexoses into cells	
*AAP1*	Amino acid permease 1			Arabidopsis	Overexpression					Increase amino acid transport into cell	^104^
*AAP6*	Amino acid permease 6			Arabidopsis	Overexpression					Increased uptake of amino acids into cell	^104^
*LIN5*	Apoplastic invertase 5	Tomato	CaMV35S	Tomato	Knockdown	-25 to -50%		− 12 to − 13%	Fruit (number and size)		^78^^;^^80^
				Tomato	QTL			Increase	Sugar Yield		
*CAT9*	Cationic amino acid transporter 9	*Arabidopsis thaliana*	*Ubiquitin*	Arabidopsis	Overexpression	1.4 (fold change)	Leaves	100%	Biomass		^105^
*INVINH1*	Apoplastic invertase inhibitor	Tomato	CaMV35S	Tomato	Overexpression			− 20%	Seed weight		^74^
*SUS1*	Sucrose synthase 1	Potato	S7 promoter from subterranean clover stunt virus	Cotton	Overexpression	2 (fold change)	Fiber length (20 DAA)	30%	Seed weight		^106^
*AgpL1*	Large subunit of ADPglucose pyrophosphorylase 1	Tomato		Tomato	Introgression line which harbored the allele from wild specie *S. Habrocjaites*	2 (fold change)	Fruits	2–15%	Fruit (fresh weight)		^107^
*TMT1*	Tonoplast monosaccharide transporter 1	*Arabidopsis thaliana*	*CaMV35S*	Arabidopsis	Overexpression			12–22%	Seed weight		^108^
*STP6*	Sugar transporter 6	*Arabidopsis thaliana*		Arabidopsis	Mutant					Increase uptake capacity of hexoses into cells	^109^
*STP3*	Sugar transporter 3	*Arabidopsis thaliana*		Arabidopsis						Increase uptake capacity of hexoses into cells	^110^

Genes have been selected based on published characterization or relation with positive effects on source, transport or sink carbon nitrogen flow. Its single relation is shown through type of intervention, species and its effect on yield, or its interpretation based on the reference cited.

**Figure 1:**
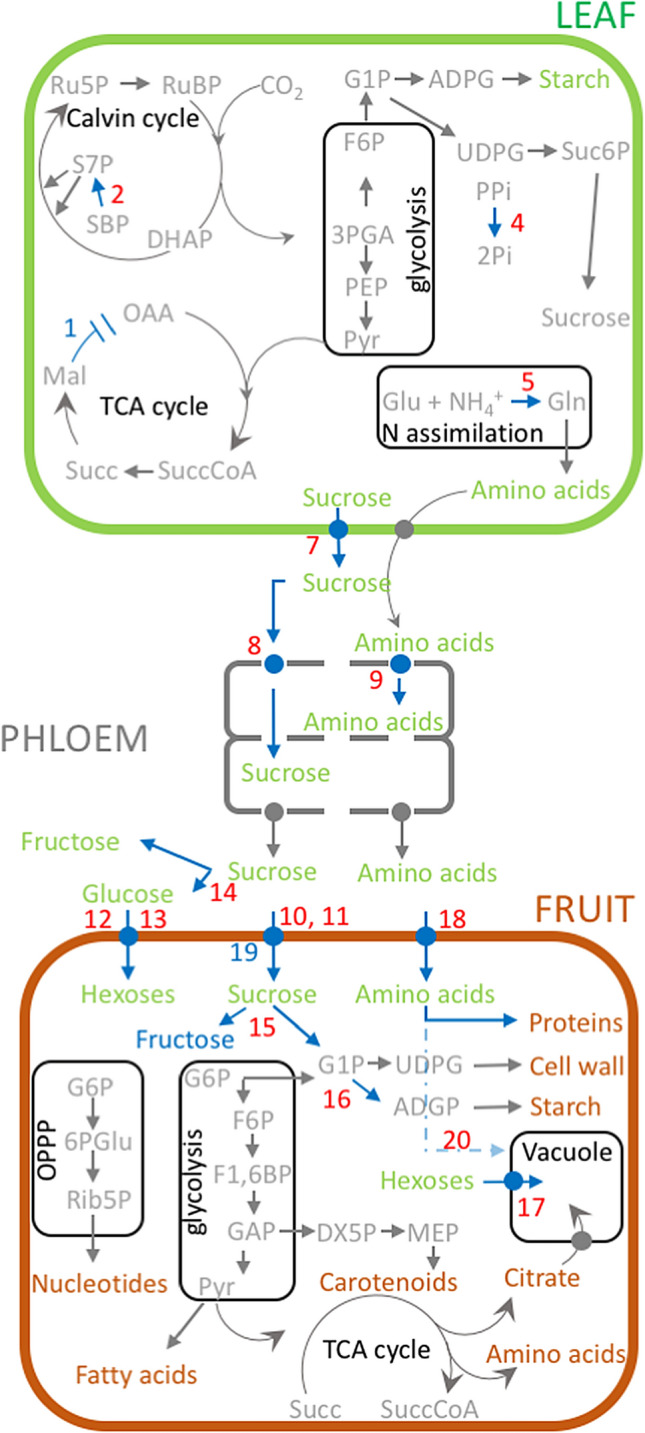
Schematic overview of stable combinatorial-transformation of tomato plants to simultaneously introduce multiple genes under different promoters to confer appropriate tissue specificity. Transgenes are involved in three different processes of carbon and nitrogen fluxes. (i) assimilation ([1] *SlmMDH, Solanum lycopersicum* mitochondrial malate dehydrogenase; [2] *AtSBP, Arabidopsis thaliana* sedoheptulose 1,7-bisphosphatase; [3] *SlSPA, Solanum lycopersicum* sugar partitioning affected; [4] *EcPP, Escherichia coli* pyrophosphatase; [5] *NtGS2, Nicotiana tabacum* chloroplast glutamine synthetase 2; [6] *FpGLDH, Flaveria pringlei* H-protein of glycine decarboxylase); (ii) transport ([7] *AtSWEET11, Arabidopsis thaliana* sugar efflux transporter 11; [8] *AtSUC2, Arabidopsis thaliana* sucrose transporter 2; [9] *AtAAP1, Arabidopsis thaliana* amino acid permease 1); and (iii) sink metabolism ([10,11] *AtSUC2/9, Arabidopsis thaliana* sucrose transporter 2/9; [12, 13] *AtSTP3/6, Arabidopsis thaliana* sugar transporter 3/6; [14] *SpLIN5, Solanum pennellii* tomato apoplastic invertase 5; [15] *AtSUS1, Arabidopsis thaliana* sucrose synthase 1; [16] *ShAgpL1, Solanum habrochaites* Large subunit of ADPglucose *pyrophosphorylase* 1; [17] *AtTMT1, Arabidopsis thaliana* tonoplast monosaccharide transporter 1; [18] *AtAAP6, Arabidopsis thaliana* amino acid permease 6; [19] *SlINVINH, Solanum lycopersicum* apoplastic invertase inhibitor; [20] *SlCAT9, Solanum lycopersicum* cationic amino acid transporter 9). Overexpression (showed as red color) or silencing (showed as blue color) of these genes were achieved using different tissue-specific promoters; (i) leaf- and mesophyll-specific, ribulose-bisphosphate carboxylase (RbcS), and fructose-1,6-bisphosphate (cyFBP); (ii) constitutive, 35S-cauliflower mosaic virus (35S); (iii) companion cell-specific, commelina yellow mottle virus (CoYMV); (iv) fruit specific, patatin B33 (B33), and ripening-specific ethylene-inducible E8 (E8); and (v) native promoter of *S. habrochaites* Large subunit of ADPglucose *pyrophosphorylase* 1 (ShAgpL1). Transgenic lines were grown under glasshouse and polytunnel conditions. *Sl*SPA resides in the plastid but is not known to catalyze an enzymatic reaction, GLDH is associated to the inner mitochondrial membrane where it catalyzes the terminal reaction of ascorbate biosynthesis.

We performed stable co-transformation of tomato plants (cv. MoneyMaker) to simultaneously introduce multiple genes under control of different promoters to confer appropriate tissue specificity (Fig. 1, Supplementary Table S1; Supplementary Note). Using an established combinatorial biolistic co-transformation protocol we were able to generate a total of 18 primary transformant lines (T_0_), which were grown in the greenhouse to produce seeds (T_1_). The T_1_ seeds were germinated on kanamycin-containing media to select for hetero- and homozygous plants. Additionally, the T_1_ plants were fully genotyped by polymerase chain reaction (PCR) assays using transgene-specific primers that do not amplify the endogenous gene. As result, a different combination of transgenes was inserted in each independent transgenic line is shown in Supplementary Table S1.

We selected three to ten T_1_ plants per line to be grown under two different growth conditions; (1) glasshouse under low light (< 450 µmol (photons) m^−2^ s^−1^ of Photosynthetically Active Radiation—PAR) and limited soil (*i.e.* pots contained approx. 0,004 m^3^ of substrate), and (2) polytunnel (semi-commercial conditions) under high light (> 1200 µmol (photons) m^−2^ s^−1^ of PAR) and non-limited soil. Initially, we set up an extra experiment under glasshouse conditions in which tomato plants were allowed to develop naturally (i.e. only side shoots were removed), however we observed that some fruits did not reach ripe stage in all transgenic plants and the two controls. Therefore, we decided to work with pruned plants to standardize and directly compare both grown conditions. Thus, all plants were pruned one week after fruit set to five fruits/truss and three trusses per plant. In addition, due to the normal early fruit-set of the first fruit of each truss, this fruit was removed in order to synchronize growth of fruits in the same truss.

### Overview of the changes in carbon- and nitrogen-related genes under low and high light and limited and non-limited soil growth conditions and in different organs

**Figure 2:**
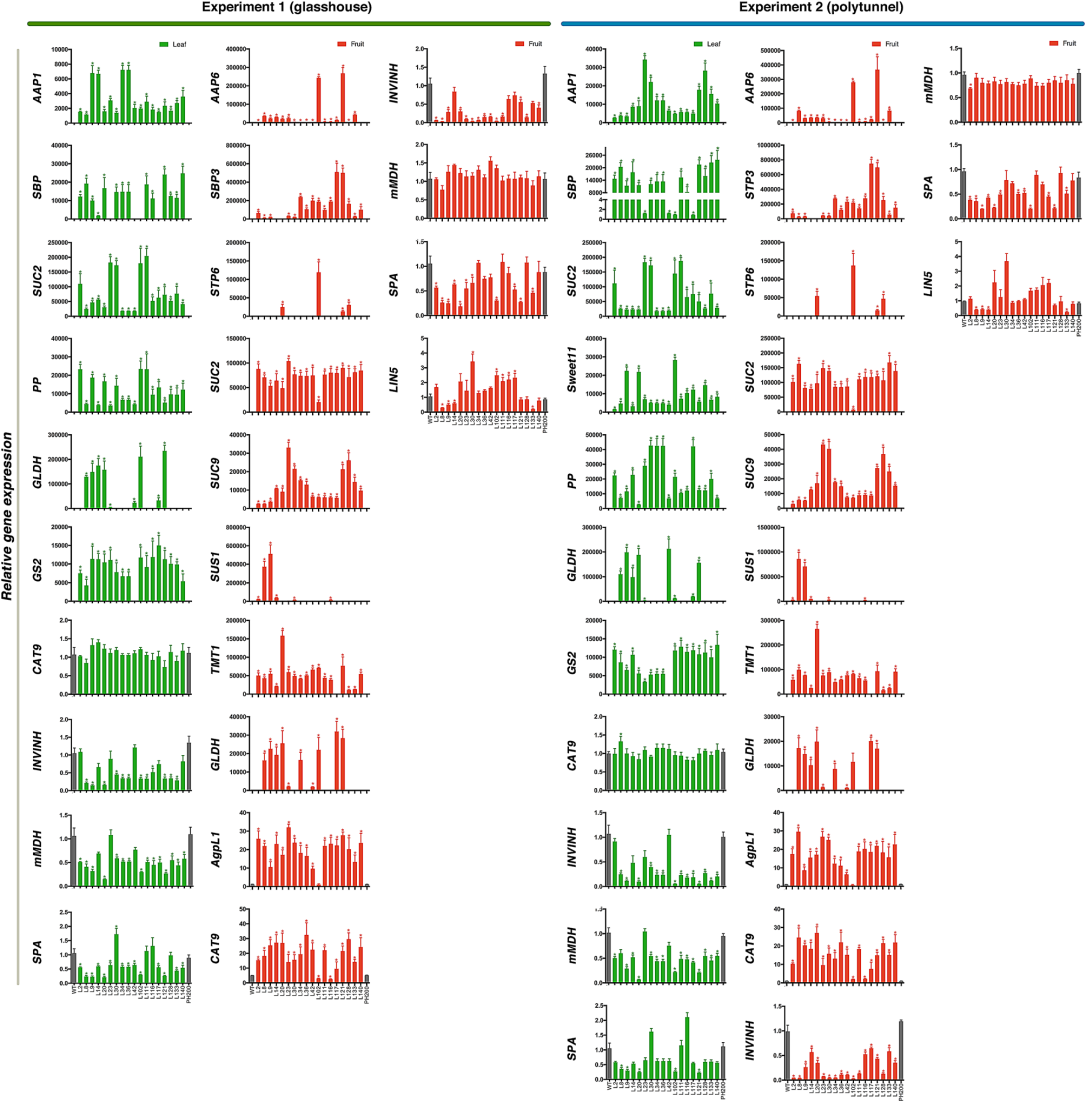
Gene expression of genes involved in carbon and nitrogen fluxes. Expression by quantitative real-time PCR (qRT-PCR) of *AAP1, SBP, SUC2, PP, GLDH, GS2, CAT9, INVINH, mMDH, SPA, AAP6, SBP3, STP6, LIN5, SUC9, SUS1, TMT1*, and *AgpL1* genes in transgenic lines under glasshouse and polytunnel conditions in fully expanded leaves and mature red fruits. The increase or decrease in expression of each gene is shown relative to the control value. Error bars indicate means ± SD. Asterisks indicate the values that were determined by the *t*-test to be significantly different (P < 0.05) from control. Note the different axes scale in the independent plots. This data is plotted with the individual data points visible in Supplementary Table S8.

In order to explore the changes in the level of transcription of all transferred genes related to carbon and nitrogen fluxes, we evaluated the relative abundance of all studied transcripts by qRT-PCR in fully expanded leaves from 4 week-old plants and mature red fruits from plants grown in the greenhouse and the polytunnel (Fig. 2). From these analyses, we confirmed that there was a reduction or overexpression of the target gene transcript restricted to tissue specificity expected for the promoter used. It is, however, important to note that a few lines showed changes in gene expression not related to the transgene (for example *SBP3* expression was increased in lines 23, 34, 42, 102, and 117 in comparison to control), although these lines were not transformed with this target gene (Supplementary Table S1). In both tissues, gene transcript levels displayed similar patterns of changes in both glasshouse and polytunnel grown conditions (Fig. 2). Effect of growth conditions and genotypes (lines) on gene expression is shown as Supplementary Data and Supplementary Table S2.

### Detailed phenotypic analysis of transgenic lines under low light, limited soil and high light, non-limited soil grown conditions

To further characterize these lines, we first performed a detailed phenotypic analysis of the plants grown either in glasshouse or polytunnel conditions. Phenotypic variation in terms of photosynthesis, dark respiration, stomatal conductance, chloroplast electron transport rate (ETR) was measured prior to flowering. In general, variation of these traits were largely similar between the growth conditions. However, large variability was evident in some lines for some traits when comparing the growth conditions (Supplementary Figure S1). In particular, we observed a decrease in (1) photosynthesis in lines 42 and 116; (2) dark respiration in lines 14, 23, 102, and 121; (3) ETR in lines 8, 42, 116, and 128 when comparing with control plants (Supplementary Fig. S1).

When analyzing fruit ripening-related traits, five lines (in particular, lines 8, 30, 111, 117 and 121) flowered significantly earlier than their respective controls in the glasshouse or polytunnel, respectively (Supplementary Figure S2A,B). Moreover, as would perhaps be anticipated, the same lines produced red fruit earlier than controls. By contrast, some lines displayed later flowering time in comparison to controls (Supplementary Figure S2C,D). Namely, when plants were grown in the polytunnel, the late flowering of lines 2 and 42 correlated with a later appearance of the first red fruit. Similarly, lines 128 and 140 showed the same behavior in the greenhouse (Supplementary Figure S2D). We next determined yield parameters of mature fruit. In glasshouse, two transformants (lines 111 and 116) displayed mild reductions in fruit yield, however it is important to note that four lines (lines 14, 36, 102, and 121) showed a significantly increased fruit yield ranging from 13.5 to 23% (Table 2). Interestingly, when transformants were grown in the polytunnel the same behavior was observed for these lines but also for lines 117 and 133 (Table 2). Moreover, the lines showing higher yield also exhibited a clear increase in the total soluble solids (Brix) content of their fruits (Table 2). By contrast, the same lines displayed unaltered or even mild decreases in Brix content when grown in the glasshouse.

**Table 2:** Total fruit yield and soluble solid content (°Brix index) of transgenic lines in comparison with the control under glasshouse and polytunnel conditions.

Line	Growth condition					
	Glasshouse (Experiment 1)			Polytunnel (Experiment 2)		
	Yield (gr/plant)	PC (%)	°Brix	Yield (gr/plant)	PC (%)	°Brix
PH200 (Control)	766.02 ± 87.2^ns^	0.0	3.46 ± 0.12^ns^	1474.08 ± 116.4^ns^	0.0	3.19 ± 0.09^ns^
2	661.70 ± 125.4^ns^	− 13.6	3.31 ± 0.23^ns^	1245.28 ± 112.8*	− 15.5	3.16 ± 0.15^ns^
8	791.07 ± 64.9^ns^	3.3	3.24 ± 0.14^ns^	1492.65 ± 33.6^ns^	1.3	3.17 ± 0.12^ns^
9	784.83 ± 74.1^ns^	2.5	3.29 ± 0.09^ns^	1576.75 ± 222.0^ns^	7.0	3.20 ± 0.13^ns^
**14**	875.96 ± 90.1*	**14.4**	3.41 ± 0.16^ns^	1695.11 ± 120.5*	**15.0**	3.37 ± 0.16^ns^
20	748.03 ± 109.6^ns^	− 2.3	3.33 ± 0.20^ns^	1354.38 ± 64.4*	− 8.1	3.19 ± 0.10^ns^
23	746.01 ± 61.4^ns^	− 2.6	3.26 ± 0.18^ns^	1450.40 ± 149.1^ns^	− 1.6	3.16 ± 0.04^ns^
30	694.96 ± 86.1^ns^	− 9.3	3.42 ± 0.12^ns^	1450.63 ± 137.7^ns^	− 1.6	3.15 ± 0.17^ns^
34	802.39 ± 55.8^ns^	4.7	3.35 ± 0.13^ns^	1573.22 ± 148.4^ns^	6.7	3.30 ± 0.15^ns^
**36**	869.62 ± 72.5*	**13.5**	3.32 ± 0.21^ns^	1706.18 ± 169.3*	**15.7**	3.35 ± 0.20*
42	693.72 ± 144.3^ns^	− 9.4	3.23 ± 0.28^ns^	1191.30 ± 114.7*	− 19.2	3.19 ± 0.03^ns^
**102**	942.07 ± 167.2*	**23.0**	3.46 ± 0.13^ns^	1724.39 ± 129.9*	**17.0**	3.41 ± 0.20*
111	650.07 ± 55.1*	− 15.1	3.26 ± 0.14^ns^	1464.25 ± 63.8^ns^	− 0.7	3.30 ± 0.07*
116	593.62 ± 113.6*	− 22.5	3.14 ± 0.23^ns^	1190.07 ± 159.8*	− 19.3	3.09 ± 0.21^ns^
117	757.00 ± 145.8^ns^	− 1.2	3.31 ± 0.18^ns^	1693.27 ± 124.7*	**14.9**	3.32 ± 0.08*
**121**	884.60 ± 72.8*	**15.5**	3.37 ± 0.18^ns^	1696.78 ± 171.6*	**15.1**	3.40 ± 0.14*
128	721.07 ± 188.4^ns^	− 5.9	3.32 ± 0.02^ns^	1425.20 ± 157.7^ns^	− 3.3	3.11 ± 0.07^ns^
133	685.66 ± 138.4^ns^	− 10.5	3.36 ± 0.07^ns^	1689.65 ± 111.5*	**14.6**	3.22 ± 0.12*
140	750.14 ± 114.8^ns^	− 2.1	3.31 ± 0.16^ns^	1573.70 ± 177.2^ns^	6.8	3.17 ± 0.13^ns^

Values are presented as means ± Sdev. Asterisks indicate values determined by Student´s *t* test to be significantly different from the control value (*p* < 0.05) and are set in bold face.

*Ns* non significant, *PC* percentage change.

### Metabolite profiling reveals differential metabolic responses to light and soil growth conditions

In order to gain a deeper understanding of the metabolic changes underlying the above-mentioned increased yield in the transgenic lines (glasshouse [experiment 1], lines 14, 36, 102 and 121; polytunnel [experiment 2], lines 14, 36, 102, 117, 121, 133), we next determined metabolite levels in the pericarp tissue of mature fruit harvested from plants grown under both growth conditions using a gas chromatography-time of flight-mass spectrometry (GC–TOF–MS)-based metabolite profiling method. A total of 47 primary metabolites were annotated after this analysis and their relative levels were normalized of each sample for each grown condition (Supplementary Tables S3 and S4). In addition, metabolite levels were analyzed on a dry weight basis to avoid the effect of differential water contents.

Each dataset was examined by principal component analysis (PCA) (Supplementary Figure S3). For fruits from plants grown in the glasshouse (experiment 1), clear differences were evident between the analyzed genotypes. However, for fruits of the high light, non-limited soil growth conditions (polytunnel; experiment 2) PCA clearly separated the genotypes along PC2, with the exception of line 121 that was separated along PC1. Overall the global composition changes induced in mature fruit in experiment 2, high light and non-limited soil grown conditions (polytunnel), seem lower than those recorded in experiment 1 (glasshouse).

**Figure 3:**
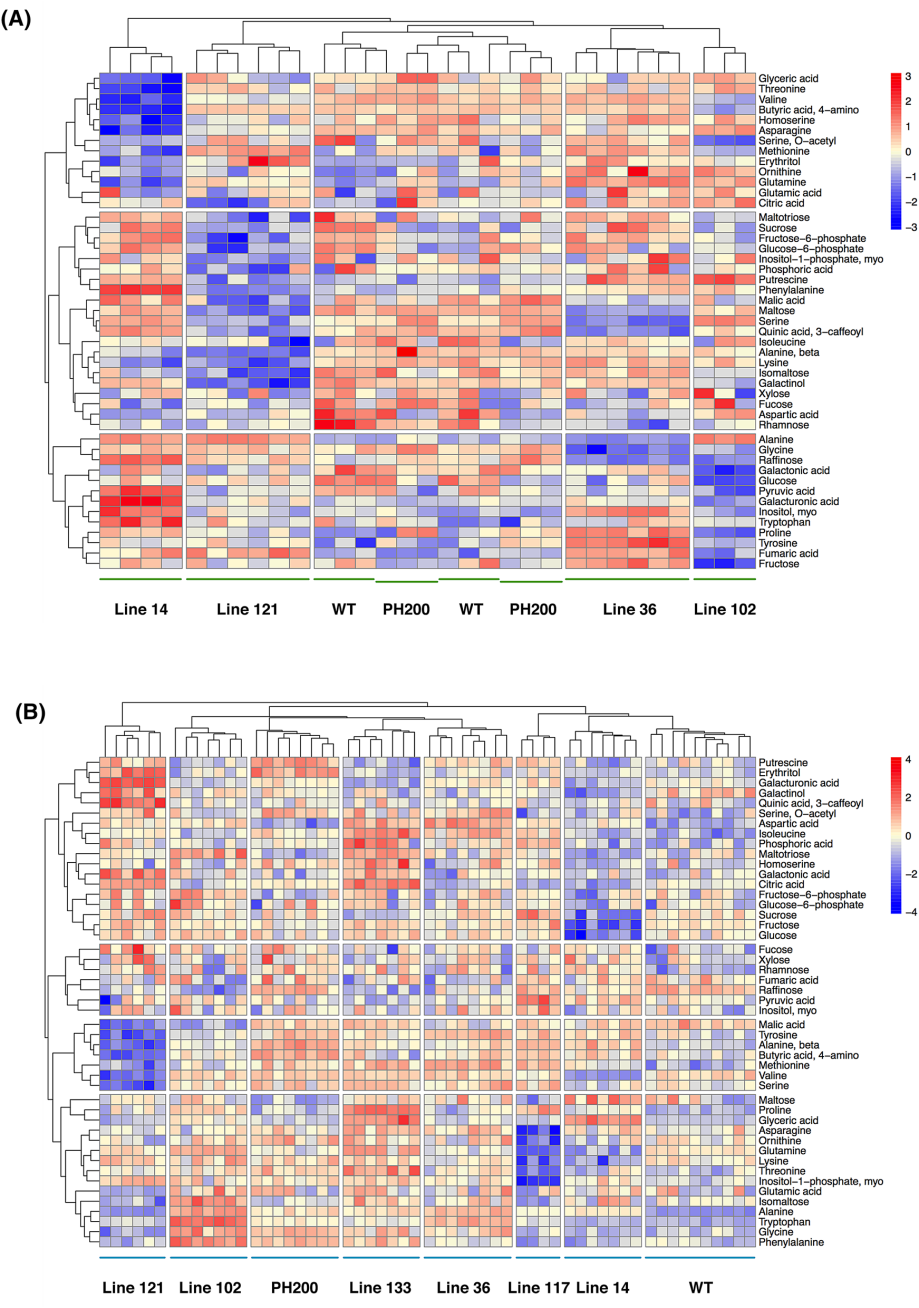
Hierarchical clustering of the primary metabolite data from selected transgenic lines under glasshouse (**A**) and polytunnel (**B**) conditions. Relative metabolite levels were normalized (Z-Score) of each sample for each grown condition and to dry weight. Each biological replicate is shown independently. For negative controls, WT and PH200 were used (PH200 was originated from an independent transformation, containing only the *nptII* gene under 35S promoter). Full documentation of metabolite profiling data acquisition is provided in Supplementary Table S3 and S4. Data analysis and graphical representation were performed using R Software (https://www.R-project.org/).

**Figure 4:**
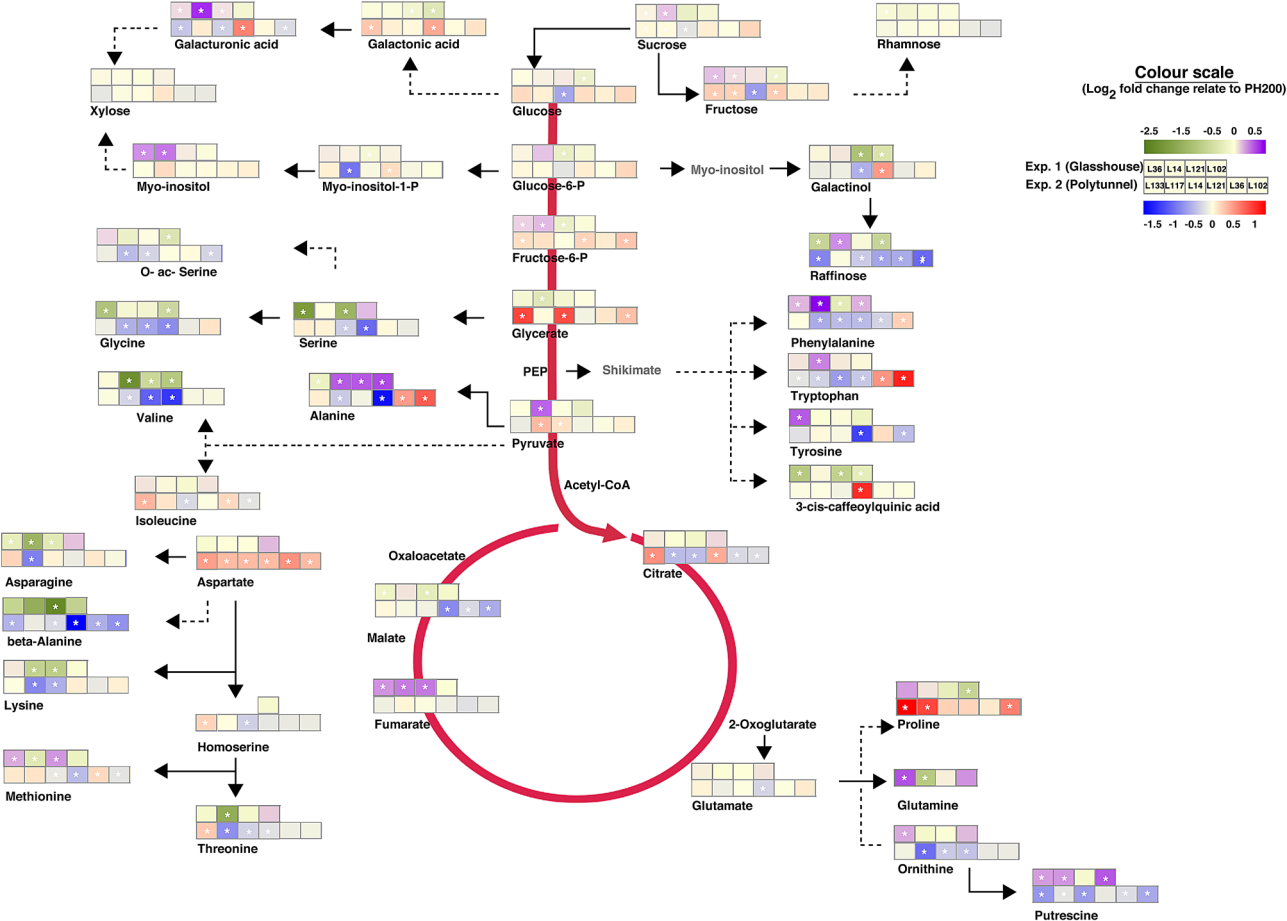
Schematic representation of metabolite changes occurring in selected transgenic lines. The heat maps represent the Log_2_ of the fold change level of metabolites with respect to the control in plants under glasshouse (violet-green) and in polytunnel (red-blue) conditions. Changes that were significant in the statistical analysis are denoted with an asterisk. The lines have been ordered by yield increase (Table 2).

The effects of the genetic intervention on the levels of individual metabolites are summarized in Supplementary Tables S3 and S4. Of the compounds analyzed, approximately 50% were significantly altered in experiment 1 (glasshouse) while more than 80% were significantly altered in experiment 2 (*p* < 0.05) (Fig. 3). Some metabolites showed a clear tendency of differential accumulation across both experiments. For example, glutamine, methionine, alanine, and putrescine accumulated in both experiments while others such as malic acid, lysine, and valine decreased (Figs. 3 and 4). Under low light and limited soil conditions (experiment 1, glasshouse), sucrose, glucose, fructose, rhamnose, galactonic acid, and proline were reduced in the high yielding transgenics in comparison to the control line. By contrast, these metabolites accumulated under in high light and non-limited soil conditions (experiment 2) in the high yielding transgenics in comparison to the control line. Decreased contents of phenylalanine and glycine were observed under both conditions, whereas ß-alanine was decreased only in polytunnel grown transgenics. Moreover, increased contents of aspartic acid, citric acid, tryptophan and isoleucine were observed solely in transgenic plants grown in polytunnel conditions.

**Figure 5:**
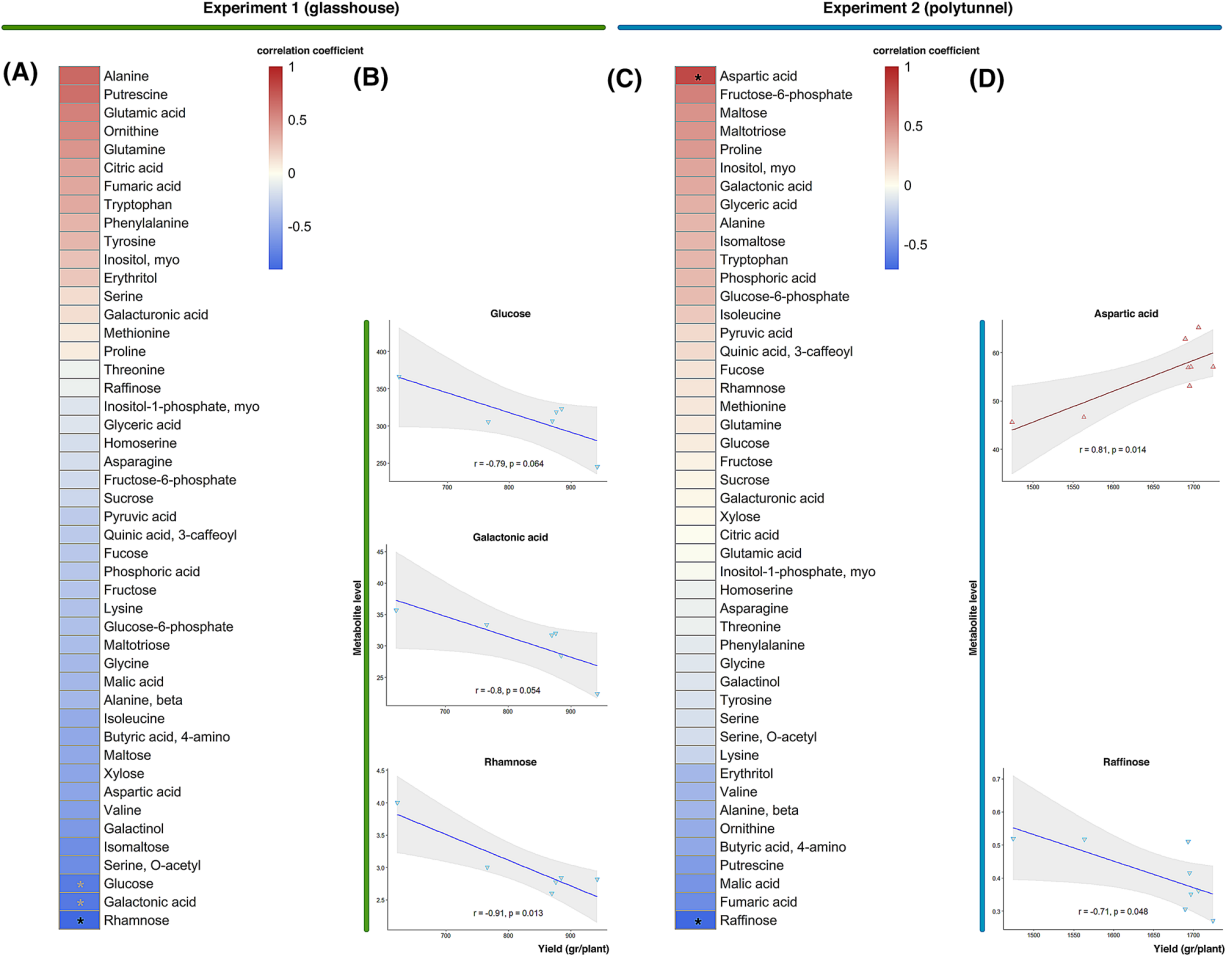
Correlation between metabolite levels and fruit yield under (**A**) glasshouse and (**B**) polytunnel conditions. Levels of selected metabolites showing significant correlation (*p* < 0.05) were plotted (**B**) and (**D**) against fruit yield. Correlation coefficient and *p*-value were calculated based on Pearson correlation analysis. Data analysis and graphical representation were performed using R Software (https://www.R-project.org/).

We next investigated the strength of correlations (based on Pearson correlation coefficients at the threshold of *p* < 0.05) between the levels of each metabolite and fruit yield in either the glasshouse or polytunnel experiment. We postulate that this would allow us to identify metabolites closely related to fruit yield under the different growth conditions. In the polytunnel grown plants levels of aspartic acid displayed a positive correlation while raffinose displayed a negative correlation to fruit yield (Fig. 5, Supplementary Table S5). Under glasshouse condition, levels of rhamnose and galactonic acid displayed negative correlation with fruit yield (Fig. 5, Supplementary Table S5). This finding suggests that these metabolites are possible candidate metabolite biomarkers related to fruit yield and highlights that the key points of regulation vary depending on the environmental conditions.

### Sparse partial least squares (sPLS) regression modeling can predict fruit yield from a combination of transcript levels

We next constructed a sparse Partial Least Squares (sPLS) regression model in order to ascertain if we could identify genes that could highly affect fruit yield in each growth condition (glasshouse and polytunnel) and also distinguish leaf and fruit tissues^55^. The model is creating variable importance in the projection (VIP) coefficients of the relative importance of each independent variable (in this instance the gene expression levels measured in this study Fig. 2, Supplementary Fig. S4), for each dependent variable (yield) of every single combinatorial experiment. In other words, the greater the VIP coefficient the greater the explanatory power with regard to yield. The model was applied to data coming from each growth condition (experiment 1; glasshouse and experiment 2; polytunnel) as well as to distinguish variables from different tissues (leaves and fruits). We ran three independent simulations for the leaves, fruits and the combination of leaves and fruits, respectively (Table 3).

**Table 3:** Sparse Partial Least Squares (sPLS) regression model applied on the gene expression values (Fig. 2) to elucidate their explanatory power resolving in fruit yield values under glasshouse and polytunnel conditions on full-expanded leaves and mature red fruits.

		sPLS's variable importance in projection (VIP)—coefficients															
		Glasshouse (Experiment 1)									Polytunnel (Experiment 2)						
	Data matrix:	Leaf and fruit			Leaf			Fruit			Leaf and fruit			Leaf			Fruit
	C.D	0.713			0.564			0.617			0.802			0.644			0.541
Tissue	Gene																
Leaf	***mMDH***	0			0						0			**1.32**			
	*SBP*	0			0						0			0			
	***SPA***	**3.50**			**3.13**						**1.88**			**1.78**			
	***PP***	0			0						**2.45**			**1.74**			
	*GS2*	0			0						0			0			
	*GLDH*	0			0						0			0			
	*Sweet11*										0			0.20			
	*SUC2*	0			0						0			0			
	***INVINH***	0			0						**2.40**			**2.21**			
	*CAT9*	0			0						0			0			
	***AAP1***	0.53			0.48						0			0			
Fruit	***mMDH***	0						0.93			**1.29**						**1.38**
	***SPA***	**1.72**						**1.93**			**1.22**						0.95
	*GLDH*	0						0.32			0.99						**1.22**
	***SUC2***	**1.89**						**2.03**			0.56						**1.49**
	*STP6*	0						0			0						0
	*STP3*	0						0			0						0.30
	***LIN5***	0						0			**1.70**						**1.61**
	*INVINH*	0						0			0						0.60
	*SUS1*	0						0			0						0.41
	*AgpL1*	0						0			0						0.63
	*TMT1*	0						0			0						0.61
	***AAP6***	**2.23**						**2.22**			**2.52**						**2.06**
	***CAT9***	0						0.49			0						**1.24**
	***SUC9***	0						0			0.11						0.73

Values represent sPLS's Variable Importance in Projection (VIP)—coefficients. Threshold for significative value has been arbitrary fixed in 1.2 and coefficients above this limit are set in bold face.

In leaves, we identified that the SPA protein contributed most significantly to variation of fruit yield under low light and limited soil (glasshouse). In addition to this protein, we also observed that pyrophosphatase and the invertase inhibitor were highly significant contributors for describing the variation in yield under high light and non-limited soil conditions (polytunnel) (Table 3).

When estimating the VIP coefficients in fruit, a total of three (under glasshouse conditions) and six (under polytunnel conditions) proteins displayed high VIP values, suggesting the significant contribution of these proteins to explain fruit yield variation under the two different grown conditions, respectively (Table 3). These proteins are: sugar partitioning affecting protein (SPA), sucrose transporter 2 (SUC2), and amino acid permease 6 (AAP6) for glasshouse conditions, and mitochondrial malate dehydrogenase (mMDH), H-protein of glycine decarboxylase (GLDH), sucrose transporter 2 (SUC2), amino acid permease 6 (AAP6), apoplastic invertase 5 (LIN5), and cationic amino acid transporter 9 (CAT9) for polytunnel (Table 3).

Furthermore, when calculating the VIP coefficients in the joint dataset (leaves and fruits combined), we observed that a large proportion of the enzymes contributing to the variation of fruit yield could be explained by the additive effects of the individual analysis for each tissue (Table 3). This confirms the importance of the expression of SPA, pyrophosphatase and the invertase inhibitor in leaves and LIN5 and AA6 in fruits Moreover, the modeling of the combined data set highlighted two transporters, amino acid permease 1 (AAP1), and sucrose transport 9 (SUC9), that also exhibited significant contribution to explain fruit yield variation only under glasshouse condition (Table 3). Whilst on the basis of the current study we cannot formally state if the variation in gene expression and enzyme activity lies in the genetic diversity or in the genotype-environment interaction, it is evident that the three processes of assimilation, transport, and sink metabolism are important in determining the fruit yield.

## Discussion

Current agriculture faces a considerable challenge with respect to securing food for the growing population on the planet, a fact that is exacerbated by the deteriorating environment and increasing pressure for land use. It is, therefore, becoming imperative to develop strategies which enable us to substantially increase crop yields on existing farmland^56^. Numerous studies have shown that partitioning and allocation of C and N assimilates play an essential role in crop yield. Considering that source-sink partitioning is determined by the synchronization of a highly complex signaling network that also embraces developmental processes^12^, there is a substantial interest in the engineering of key metabolic processes for increased C and N flow. Several published studies have determined that high availability of C sources leads to higher C accumulation on the sink^57,58^. However, there are also a number of previous studies of sink-dependent alteration of photosynthesis of source leaves by using single-transgene transformation^59–63^. This suggests that the photosynthetic activity of source tissues is controlled either by the metabolism of photoassimilates within source tissue, insufficient sink strength or inhibition of their transport^64^. This hypothesis is further supported by experiments in potato and pea which indicate that transgenic manipulation of both source and sink is a highly effective route for enhancing the harvest index of a crop species^39,48^. Recently, a multi-transgenic approach has been used that targeted both C and N metabolism was proven to be effective in enhancing Arabidopsis growth^65^. Our study expands on the basis of those above by generating multi-transgenic tomato plants that are affected in both source and sink metabolism to simultaneously increase the flow of C and N from leaves to fruit with a view to altering yield. The aim of this work was to determine the importance of twenty proteins previously implicated (see the summary in Table 1), in diverse processes of source-sink partitioning, in the reconfiguration of plant metabolism required to increase fruit yield.

In search of the combination with the greatest impact on yield, we expressed different genes under diverse promoters in order to achieve a range of protein overexpression or silencing. For overexpression, to achieve high expression levels, we used the CaMV 35S viral promoter which has been widely and successfully used in the past to drive high expression of transgenes^66^. In addition, RbcS, cyFBP, CoYMV, Patatin B33, and E8 promoters allowed us to achieve intermediate level expression and leaf-, mesophyll-, companion cell-, fruit- and fruit ripening- specific expression, respectively. For gene silencing, either the RbcS or the CoYMV promoter was used. We subsequently evaluated the physiological and metabolic effects of these genetic interventions under two different grown conditions, (1) glasshouse under relative low light (< 450 PAR) and limited soil (pots contained approx. 0.004 m^3^ of substrate), and (2) polytunnel (semi-commercial conditions) under high light (> 1200 PAR) and non-limited soil.

We observed common transgenic lines (namely L14, L36, L102, L121) exhibiting significantly increased fruit yield in our experiments under both low light, limited soil conditions and high light, non-limited soil growth conditions. In addition, two more transgenic lines (L117 and L133) displayed elevated fruit yield in comparison to control plants under high light, non-limited soil conditions. That said, the rest of transgenic lines did not display consistent differences across the experiments rendering it difficult to associate phenotypic and metabolic characteristics of these plants with fruit yield. Focusing exclusively on the transgenic lines displaying increased fruit yield, we observed that these plants produced heavier fruits although the number of fruits were identical since the plants had previously been pruned. Moreover, neither morphological not developmental alterations appeared under both grown conditions (greenhouse and polytunnel). Given the lack of significant alteration in photosynthetic parameters our results indicate a more efficient transfer of photoassimilate between source and sink. This hypothesis was supported by the analysis relating gene expression and fruit yield by applying a sparse Partial Least Squares (sPLS) regression model on leaves and fruits separately. When the transcript levels relation was tested in leaves under low light, limited soil grown conditions, we found that only the expression of the *Sugar Partitioning-Affecting (SPA)* gene*,* exhibited a high VIP value with fruit yield. Our analysis is in line with the observation that deficiency of this protein, which is encoded by a single gene in tomato^67^, leads to a pronounced phenotype, with increased harvest index and reduction in the level of sucrose, glucose and fructose in leaves^68^. These changes indicate that SPA activity promotes carbon export from leaves to sink organs. Interestingly, under the same grown condition, when we tested the regression model on fruit, expression of *SUC2* and *AAP6* genes appeared to be important, in addition to *SPA,* to explain higher fruit yield under low light, limited soil grown conditions. APP6 has been described to play a role in xylem-phloem transfer^69^. This hypothesis is supported by showing a reduction in amino acid contents of sieve elements in *aap6* mutant in Arabidopsis^70^. Moreover, this mutant did not display a strong phenotype, only a slight increase in leaf width and seed size. Interestingly, the third candidate gene highlighted from the model was *SUC2*, an apoplastic loader, stressing the importance of sugar movement system across the plasma membrane for phloem loading to increase fruit yield. In particular, sucrose is loaded into the sieve element-companion cell complex in the phloem by the sucrose-H^+^ co-transporter SUC2 from the apoplasm (cell wall space)^71^. Interestingly, potato plants that expressed reduced levels of this sucrose transporter showed a dramatic reduction in tuber yield, supporting the importance of transport capacity for growth and development of the plant^71^.

When the above approach was used to identify genes that highly affect fruit yield in leaves from plants grown under high light and non-limited soil condition, we found that two proteins having a role in assimilation of carbon, soluble pyrophosphatase (PP) and in sink metabolism, apoplastic invertase inhibitor (INVINH), were identified to have high contributions to explain increased fruit yield on plants grown in polytunnel. These results pointed to the importance of increase the gradient of translocation from source to sink and hence the net import into the fruit under high light grown condition. Consistent with this hypothesis, overexpression of *E. coli* PP previously described in tobacco and potato resulted in sugar-storing leaves^72,73^—a feature which could subsequently be exploited by re-routing these photoassimilates to the sink organs^39^. In particular, transgenic lines of tobacco and potato showed perturbed sink growth but different responses. In tobacco, plant growth was inhibited, while potato plants produced a larger number of smaller tubers in comparison to controls^72,73^. In addition, Jin et al.^74^ showed that decreasing the INVINH activity in tomato correlated with an increased fruit sugar level and seed size without a negative impact on fruit yield.

Finally, a tight co-regulation of C-N metabolism was observed in fruits from plants grown under high light and non-limited soil conditions, since the combination of six protein activities (named as mMDH, GLDH, SUC2, AAP6, LIN5, and CAT9) were needed to significantly explain the increased fruit yield. In particular, these results illustrate the intertwined crosstalk of metabolic pathways through assimilation, transport, and sink metabolism of photoassimilates for the maintenance of carbon and nitrogen metabolism to increase fruit yield. In this sense, our data support the hypothesis of enhance fruit yield under high light grown condition only through a tightly coordinated increase in carbon assimilation, export, and utilization. This scenario is in agreement with previous studies in which reduced activity of mMDH detected in source leaves correlated with an induction of photosynthetic metabolism in leaves, resulting in increased fruit yield^75^; however, fruit-specific antisense suppression of this enzyme resulted in a relatively small effect on total fruit yield^76^. Moreover, using an in vitro assay, Hasse et al.^77^, demonstrated that increased glycine decarboxylase (GLD) H-protein supply enhances the activity of GLD P-protein, an essential protein for the interconversion of glycine and serine in photorespiration^78^. Furthermore, overexpression of *GLDH* resulted in an increase in photosynthesis and yield^24,79^. The present data suggest that the principal tomato phloem unloading under high light grown condition to favor an increase in fruit yield may be apoplastic through the activity of LIN5 protein as previously described^80,81^. This hypothesis is supported by the facts that reduction of LIN5 activity in tomato plants resulted in a compromised fruit yield, approximately 40% reduction of that showed for wild type^81^. CAT9 activity was also significantly identified to explain the variation of fruit yield under high light and non-limited grown conditions. CAT9 has been identified as tonoplast-localized transporter that facilities the exchange of glutamic acid, aspartic acid and GABA. This may result from the importance of GABA metabolism in signaling, redox regulation, energy production and the maintenance of carbon/nitrogen balance^82^, however, further studies are required in order to elucidate the role of this protein in the elevation of tomato yield. Another aim of this study was to identify whether there were metabolic features that rendered the transgenic lines that displayed higher yield. In this regard, we made some interesting observations further discussed in Supplementary Discussion that lead to a more complete understanding of the metabolic process in tomato to improve source-to-sink partitioning and thereby yield.

## Conclusion

The primary aim of this work was to test if a multi-step metabolic engineering of primary metabolism could be utilized to improve source-to-sink partitioning and thereby yield. For this purpose we introduced up to 20 transgenes targeted at step in source and sink metabolism as well as at the transport process itself. Under two different growth regimes we were able to identify a subset of the 20 obtained transgenic lines which had a similar magnitude of effect on yield as was achieved by single-transgene transformations but were not able to isolate lines in which the increase in yield was in excess of that previously achieved. Several possible reasons can be postulated for this however we find two of these to be most likely. Firstly, it is highly possible that we did not screen enough transgenic lines in this study to ensure that the optimal expression level of the transgenes was achieved. Secondly, it is additionally possible that our understanding of metabolism is not quite at the level whereby we can rationally “pick and mix” the best combinations of genes. It is important to note that one possible reason that we did not observe genotypes exhibiting higher yield than that achieved following single transgene manipulation was the growth space constraints in a research laboratory setting (although the growth space we utilized was considerably). As such, industrial-scale testing of this approach may allow isolate of such successful genotypes given that testing all the combinations of expression would need a vast amount of independent transformants. Since the initiation of this project a handful of elegant papers boosting tomato yield by affecting development associated genes have been published^83,84^. It seems likely that, as was recently postulated^83^, approaches incorporating both metabolic and developmental genes would be more likely to result in larger yield increases than reported here. Despite the biolistic combinatorial co-transformation approach taken here not being highly successful from a biotechnological standpoint it did provide considerable insight into source-sink partitioning. Indeed, both the physiological and metabolic measurements support the conclusion that the phloem transport step is highly important in determining source-sink relations in tomato whilst the importance of source and sink metabolism per se is more context dependent. That said under commercial growth conditions it would seem likely that all three processes co-limit tomato fruit yield.

## Methods

### Plant material

Tomato plants (*Solanum lycopersicum* cv. Moneymaker) were grown under sterile conditions on agar-solidified MS medium^85^ supplemented with 20 g/L sucrose. Genetically modified plants were propagated and rooted in the same medium additionally containing 35 mg/L kanamycin. For sampling and seed production, plants were transferred to soil and grown under experimental growth conditions.

### Experimental growth conditions

Three to ten T_1_ plants per line were cultivated under two types of semi-controlled conditions. (1) In “experiment 1”, plants were grown in a glasshouse as previously reported^86^. Plants in the “experiment 1” were exposed to low light (< 450 µmol photons m^−2^ s^−1^ of Photosynthetically active radiation-PAR) and limited soil (i.e. pots contained approx. 0.004 m^3^ of substrate) at controlled temperature 24 °C/16 °C day/night. The plants were irradiated with supplemental light to maintain an irradiance close to 400 μmol photons m^−2^ s^−1^. (2) In “experiment 2”, plants were cultivated in polytunnel conditions (similar to semi-commercial conditions), with high light (> 1200 µmol photons m^−2^ s^−1^) and non-limited soil. Plants were pruned one week after fruit set to five fruits per truss and three trusses per plant. In addition, due to the normal early fruit-set of the first fruit in each truss, this fruit also was removed in order to avoid unbalanced growth between fruits of the same truss. Systematically, every week side shoots and new flowers were removed. Young fully expanded leaves were harvested from 4 week-old-plants. The stage of fruit development was followed by tagging the truss upon appearance of the flower. Pericarp samples were harvested from mature red fruit. Harvested fruits were weighed, and pericarp was separated from the placental tissue, weighed, and then immediately frozen in liquid nitrogen before being stored at − 80 °C until further analysis.

### Construction of transformation vectors

Transformation vectors (pSKJ1, 2, 3, 6, 8, 10, 12, 15, 16, 18, 20, 22, 24, 26, 28, 30 ad 32) were constructed based on the pUC18 plasmid, containing the cauliflower mosaic virus (CaMV 35S) promoter region upstream of the multiple cloning site (MCS) and the nopaline synthase *nos* terminator sequence downstream of the MCS. Full coding sequences of genes of interest (GOI) were amplified using a standard PCR protocol from donated plasmids, amplified from cDNA as a template or synthesized commercially (GeneCust, France). GOI sequences were subcloned into the pUC18 backbone via standard restriction enzyme type IIS and ligation-based protocol. Where needed the 35S promoter sequence was exchanged for a number of tissue-specific promoters such as Commelina yellow mottle virus (CoYMV) promoter region, B33 Patatin promoter region, *Solanum tuberosum* cytosolic fructose-1,6-bisphosphatase (StcyFBP) promoter region, *Solanum lycopersicum* small subunit of Rubisco (SlRbcS) promoter region, ethylene-inducible, ripening-specific (E8) promoter region and a native promoter region of the *Solanum habrochaites* ADP-glucose *pyrophosphorylase* Large subunit 1.Silencing vectors (pSKJ33 and pSKJ35) were constructed based on the pK7GWIWG2(I) destination vector according to the Gateway cloning protocol (Supplementary Table S6). Prior to transformation all constructs were validated by sequencing and GOI sequences were confirmed.

The plasmid cocktail (pSKJcombi1) for combinatorial transformation was prepared by mixing equal quantities of pSKJ1, 2, 3, 6, 8, 10, 12, 15, 16, 18, 20, 22, 24, 26, 28, 30, 32, 33, 35 and pK7GWIWG2(I)_SlSPA^68^ (each at a concentration of 2 µg/µL) and plasmid pPH200 that contains the *nptII* gene for kanamycin resistance between the 35S promoter and terminator (Supplementary Table S6).

### Combinatorial nuclear transformation and selection of transgenic tomato plants

Young leaves from plants grown under aseptic conditions were harvested and bombarded with gold particles coated with a plasmid DNA mixture pSKJ-combi1 (Supplementary Table S6) using the DuPont PDS1000He biolistic gun as previously described by Elghabi et al.^87^. Kanamycin-resistant shoots were selected on plant regeneration medium containing 2.0 mg/L Zeatin, 0.1 mg/L IAA, 0.5 g/L MES and 35 mg/L kanamycin. Resistant shoots were rooted in agar-solidified MS medium, then transferred to soil and grown to maturity under standard greenhouse conditions. As negative controls wild type (WT) plants were used, as well as PH200 line, which contained only the *nptII* gene controlled by 35S promoter. The PH200 line, was originated from an independent transformation. Material from T_0_ plants was harvested and used for initial molecular analysis.

### Isolation of nucleic acids

Tomato leaf genomic DNA was isolated using a CTAB-based protocol^88^ and used for genotyping. For total tomato leaf RNA extraction, samples of 100 mg of frozen leaf powder material were extracted with the NucleoSpin RNA Plant kit following the manufacturer’s instructions (Macherey–Nagel, Düren, Germany. The RNA was eluted in 60 µl of RNase-free water and stored at − 80 °C until used for the cDNA synthesis. Tomato pericarp RNA was obtained using the TRIZOL reagent according to the manufacturer’s instructions. Obtained RNA was additionally purified using the NucleoSpin RNA Plant kit.

### cDNA synthesis

Isolated RNA was tested for the presence of DNA contamination by a standard PCR using 1 ng of RNA as template. cDNA was synthesized using the SuperScript III Reverse Transcriptase kit according to the manufacturer’s instructions (Invitrogen, Carlsbad, CA). The quality of the cDNA was tested by a standard PCR reaction.

### Genotyping

Genotyping of transgenic lines was performed using genomic DNA isolated from 2-week old seedlings germinated on kanamycin-containing media. Gene-specific primers were used for genotyping. Genotyping was performed using a standard PCR protocol.

### Gene expression analysis by quantitative real-time PCR (qRT-PCR)

Quantitative RT-PCR was performed in a LightCycler 480 (Roche, Mannheim, Germany) using cDNA as template in 5 µL reactions containing 1 µL of each gene-specific primer (1.25 µM; Supplementary Table S7), 2.5 µL of the LightCycler 480 SYBR green I Master mix and 0.5 µL of a 1:50 cDNA dilution. Two biological replicates (independent plants) and three technical replicates per line were analyzed. The relative transcript levels were determined using the formula (1 + E)^−ΔΔCp^ where E is the binding efficiency of the primers^89^. Expression data were normalized to the reference gene *SlFRG03* (*Solyc02g063070*) according to Cheng et al., 2017^90^.

### Metabolite analysis

Metabolite extraction, derivatization, and sample injection for gas chromatography coupled to electron impact ionization-time of flight-mass spectrometry (GC-EI-TOF/MS) were performed according to Osorio et al.^91^. Chromatograms and mass spectra were evaluated using ChromaTOF 1.0 (Leco, www.leco.com) and TagFinder v.4.0^92^, respectively Cross-referencing of mass spectra was performed with the Golm Metabolome database^93^. Data is reported following the standards suggested in Fernie et al.^94^.

### Measurement of fruit °Brix and yield

Ripe fruit tissue was homogenized with a razor blade, and the soluble solids (Brix) content of the resulting juice measured on a portable refractometer (Digitales Refrktometer DR6000; Krüss Optronic GmbH, Hamburg, Germany). Fruit yield was determined in red fruit considering each biological replicate the weight of 15 fruits per individual plant.

### Measurements of photosynthetic parameters

Leaf gas exchange and chlorophyll a fluorescence were measured simultaneously with an open infrared gas‐exchange analyser system equipped with a leaf chamber fluorometer (Li‐6400XT, Li‐Cor Inc., Lincoln, NE, USA). The measurements were performed during mornings (9:00–11:00 h) in full expanded leaves at growth light (i.e. Glasshouse: 450 µmol (photons) m^−2^ s^−1^ of PAR, and Polytunnel 1200 µmol (photons) m^−2^ s^−1^ of PAR) while the amount of blue light was set to 10% photosynthetically active photon flux density to optimize stomatal aperture. The reference CO_2_ concentration was set at 400 µmol CO_2_ mol^–1^ air. All measurements were performed using the 2 cm^2^ leaf chamber maintaining the block temperature at 25 °C and flow rate 300 mmol air min^–1^. Dark respiration and maximum quantum efficiency of *PSII* (*F*_*v*_*/F*_*m*_) were measured during mornings in leaflets after 2 h of dark adaptation. Relative electron transport rate (rETR) was calculated according to Krall and Edwards^95^. The photorespiration rate was calculated following the model based on gas exchange and Chl fluorescence measurements proposed by Valentini et al.^96^.

### Data analysis

Data mining, normalization, clustering and graphical representation were performed using R Software (https://www.R-project.org/) and pheatmap: Pretty Heatmaps. R package version 1.0.12. (https://CRAN.R-project.org/package=pheatmap). Sparse Partial Least Squares (sPLS) regression model was performed using quantitative data. In particular, the levels of transcripts as independent variables and fruit yield under glasshouse and polytunnel conditions as dependent variables. Six different matrixes were used to feed the model; i.e.: in glasshouse (experiment 1) (i) leaf, (ii) fruit gene expression and (iii) the mixed matrix considering both datasets. Same manner, the matrixes (iv), (v) and (vi) with data coming from polytunnel (experiment 2). To determine the optimal number of components and variables of a given model, we searched the parameter space spanned all possible component combinations. For each such component/variable combination, 100 iterations of fivefold cross-validation rounds were tested. One an optimal number of components and variables was determined for each response variable, we obtained the variable importance in projection (VIP) coefficients reported. This analysis was performed using the package mixOmics^97^.

## Supplementary information

Supplementary Information

Supplementary Table S3

Supplementary Table S4

Supplementary Table S5

Supplementary Table S6

Supplementary Table S7

Supplementary Table S8
