# Supplementary material for: Multi-gene metabolic engineering of tomato plants results in increased fruit yield up to 23%
Source: Sci Rep. 2020 Oct 14;10:17219. doi: 10.1038/s41598-020-73709-6 (PMC7560729; doi:10.1038/s41598-020-73709-6)
Supplement: Supplementary file 1 — Supplementary Information [file 41598_2020_73709_MOESM1_ESM.pdf]

Supplementary information

Multi-gene metabolic engineering of tomato plants results in increased fruit yield up to 23% but does not exceed the improvement obtained by single gene transformation

Jose G. Vallarino, Szymon Kubiszewski-Jakubiak, Stephanie Ruf, Margit Rößner, Stefan Timm, Hermann Bauwe, Fernando Carrari, Doris Rentsch, Ralph Bock, Lee J. Sweetlove, Alisdair R. Fernie.

Classification		Assimilation						Transport			Sink Metabolism											NA
Promoter		RcbS	RcbS	35S	cyFBP	RcbS	35S	RcbS	coYMV	coYMV	B33	B33	B33	B33	B33	coYMV	B33	Native	B33	B33	E8	NA
Gene		*SlmMDH*	*AtSBP*	*SlSPA*	*EcPP*	*NtGS2*	*FpGLDH*	*AtSWEET11*	*AtSUC2*	*AtAAP1*	*AtSUC2*	*AtSUC9*	*AtSTP3*	*AtSTP6*	*SpLIN5*	*SlINVINH*	*AtSUS1*	*ShAgpL1*	*AtTMT1*	*AtAAP6*	*SlCAT9*	*nptII*
Line	WT	(-)	(-)	(-)	(-)	(-)	(-)	(-)	(-)	(-)	(-)	(-)	(-)	(-)	(-)	(-)	(-)	(-)	(-)	(-)	(-)	(-)
	L2						(-)			(-)	(-)											
	L8										(-)											
	L9										(-)											
	L14										(-)			(-)								
	L20													(-)								
	L23		(-)								(-)						(-)					
	L30						(-)				(-)											
	L34		(-)								(-)											
	L36						(-)				(-)						(-)					
	L42		(-)								(-)						(-)					
	L102		(-)				(-)										(-)				(-)	
	L111						(-)				(-)						(-)					
	L116										(-)										(-)	
	L117										(-)						(-)		(-)			
	L121																(-)					
	L128						(-)										(-)					
	L133						(-)				(-)						(-)					
	L140						(-)				(-)						(-)					
	PH200	(-)	(-)	(-)	(-)	(-)	(-)	(-)	(-)	(-)	(-)	(-)	(-)	(-)	(-)	(-)	(-)	(-)	(-)	(-)	(-)	

Supplementary Table S1. Schematic overview showing the number and natures of transgenes inserted in each independent transgenic line. The selected genes can be classified by three different processes of carbon and nitrogen metabolism; (i) assimilation *(SlmMDH*, *Solanum lycopersicum* mitochondrial malate dehydrogenase; *AtSBP*, *Arabidopsis thaliana* sedoheptulose 1,7-bisphosphatase; *SlSPA, S.lycopersicum* sugar partitioning affected; *EcPP, Escherichia coli* pyrophosphatase; *NtGS2, Nicotiana tabacum* chloroplast glutamine synthetase 2; *FpGLDH, Flaveria pringlei* H-protein of glycine decarboxylase; (ii) transport (*AtSWEET11, A. thaliana* sugar efflux transporter 11*; AtSUC2, A. thaliana* sucrose transporter 2*; AtAAP1, A. thaliana* amino acid permease 1; and (iii) sink metabolism (*AtSUC2/9, A. thaliana* sucrose transporter 2/9; *AtSTP3/6, A. thaliana* sugar transporter 3/6; *SpLIN5, S. pennellii* tomato apoplastic invertase 5; *SlINVINH, S. lycopersicum* apoplastic invertase inhibitor, *AtSUS1, A. thaliana* sucrose synthase 1; *ShAgpL1, S. habrochaites Large subunit of ADPglucose pyrophosphorylase 1*; *AtTMT1, A. thaliana* tonoplast monosaccharide transporter 1; *AtAAP6, A. thaliana* amino acid permease 6; *SlCAT9, S. lycopersicum* cationic amino acid transporter 9. Overexpression or silencing of these genes were achieved using seven different tissue specific promoters; (i) leaf- and mesophyll-specific, ribulose-bisphosphate carboxylase (RbcS), and cytosolic fructose-1,6-bisphosphate (cyFBP), respectively; (ii) constitutive, 35S-cauliflower mosaic virus (35S); (iii) companion cell-specific, commelina yellow mottle virus (coYMV); (iv) fruit specific, patatin B33 (B33), and ripening-specific ethylene-inducible E8 (E8); and (v) native promoter of *S. habrochaites* Large subunit of ADPglucose *pyrophosphorylase 1* (ShAgpL1). NA: not applicable. Negative amplification (or no detected transgene) on gPCR results are denoted by (-).

Supplementary Table S2: Analysis of the effect of genotypes and growth conditions.

P-value of two-way (factorial) ANOVA to test the effect of the genotype (gene expression) and the condition (*i.e.* environmental condition of the two experiments carried out in glasshouse and polytunnel) on fruit and leaves matrixes. * denotes significant effects of genotype, condition or interaction of them on gene expression ($P<0.001$).

Effect Tests on Fruits Gene Expression

Response *SlmMDH*:

Source	Prob > F
genotype	0.0698
condition	<.0001*
genotype*condition	0.0062*

Response *SlSPA*:

Source	Prob > F
genotype	<.0001*
condition	<.0001*
genotype*condition	0.4814

Response *FpGLDH*:

Source	Prob > F
genotype	<.0001*
condition	0.0002*
genotype*condition	0.0888

Response *AtSUC2*:

Source	Prob > F
genotype	<.0001*
condition	<.0001*
genotype*condition	0.0023*

Response *AtSTP6*:

Source	Prob > F
genotype	<.0001*
condition	0.2121
genotype*condition	0.9826

Response *AtSTP3*:

Source	Prob > F
genotype	<.0001*
condition	<.0001*
genotype*condition	0.0003*

Response *SpLIN5*:

Source	Prob > F
genotype	<.0001*
condition	0.0503

Source	Prob > F
genotype*condition	0.9691

Response *SlINVINH*:

Source	Prob > F
genotype	<.0001*
condition	0.0164*
genotype*condition	0.8617

Response *AtSUS1*:

Source	Prob > F
genotype	<.0001*
condition	0.0005*
genotype*condition	<.0001*

Response *ShAgpL1*:

Source	Prob > F
genotype	<.0001*
condition	0.0314*
genotype*condition	0.8479

Response *AtTMT1*:

Source	Prob > F
genotype	<.0001*
condition	<.0001*
genotype*condition	<.0001*

Response *AtAAP6*:

Source	Prob > F
genotype	<.0001*
condition	0.0063*
genotype*condition	0.2390

Response *SlCAT9*:

Source	Prob > F
genotype	<.0001*
condition	0.0054*
genotype*condition	0.8607

Response *AtSUC9*:

Source	Prob > F
genotype	<.0001*
condition	<.0001*
genotype*condition	<.0001*

Effect Tests on Leaves Gene Expression

Response *SlmMDH*:

Source	Prob > F
genotype	<.0001*
condition	0.1656
genotype*condition	0.7375

Response *AtSBP*:

Source	Prob > F
genotype	<.0001*
condition	0.8979
genotype*condition	0.0002*

Response *SlSPA*:

Source	Prob > F
genotype	<.0001*
condition	0.0651
genotype*condition	0.0030*

Response *EcPP*:

Source	Prob > F
genotype	<.0001*
condition	<.0001*
genotype*condition	<.0001*

Response *NtGS2*:

Source	Prob > F
genotype	<.0001*
condition	0.9611
genotype*condition	0.0009*

Response *FpGLDH*:

Source	Prob > F
genotype	<.0001*
condition	0.1575
genotype*condition	<.0001*

Response *AtSUC2*:

Source	Prob > F
genotype	<.0001*
condition	0.2405
genotype*condition	0.9996

Response *SlINVINH*:

**Source**	**Prob > F**
genotype	<.0001*
condition	<.0001*
genotype*condition	0.0110*

Response *SlCAT9*:

**Source**	**Prob > F**
genotype	0.2167
condition	0.0096*
genotype*condition	0.0188*

Response *AtAAP1*:

**Source**	**Prob > F**
genotype	<.0001*
condition	<.0001*
genotype*condition	<.0001*

Response *AtSweet11*:

**Source**	**Prob > F**
genotype	<.0001*
condition	0.1027
genotype*condition	0.0263*

Supplementary Figure S1. Photosynthesis, dark respiration, stomatal conductance, chloroplast electron transport rate (ETR) parameters in transgenic lines measured under A) glasshouse and B) polytunnel conditions. Data presented are means ± SD (n between six and 10 plants per line). An asterisk indicates the values that were determined by the t-test to be significantly different (*P* < 0.05) from control.

<u>A) Glasshouse</u>

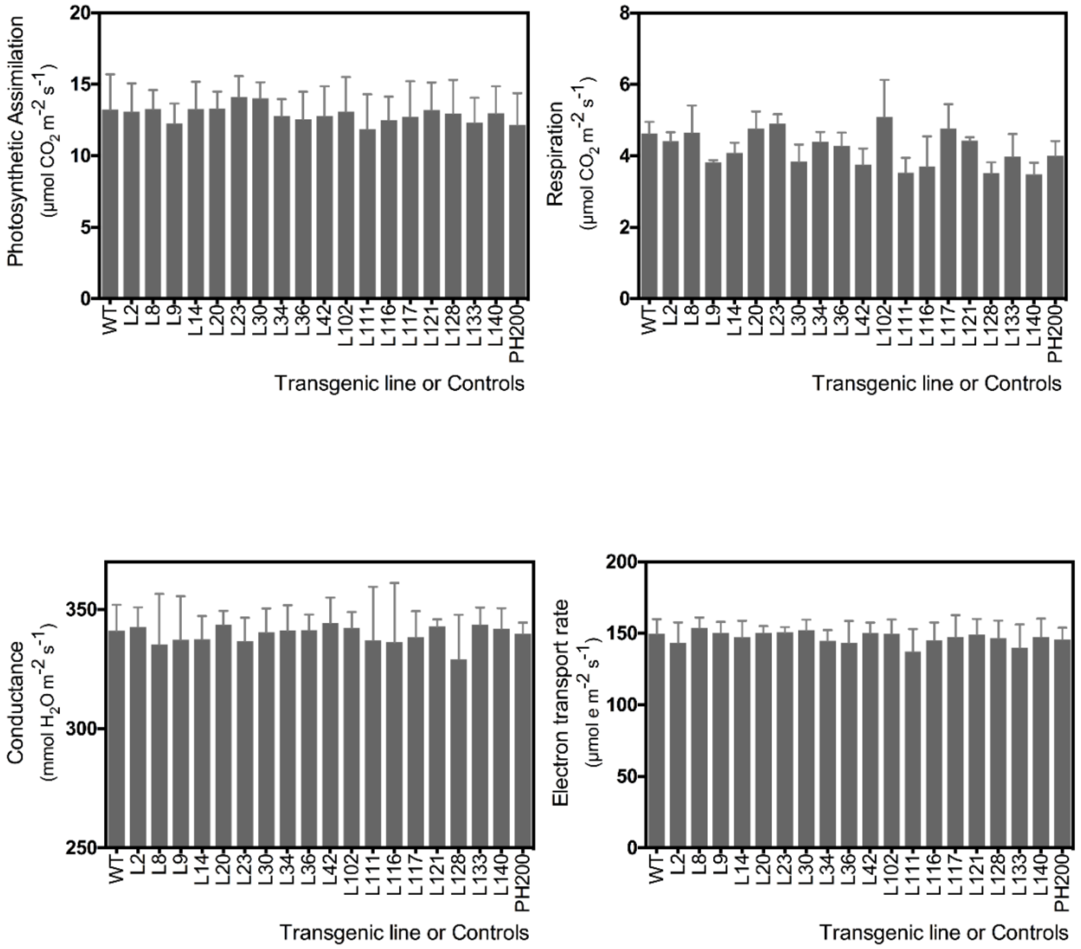

B) Polytunnel

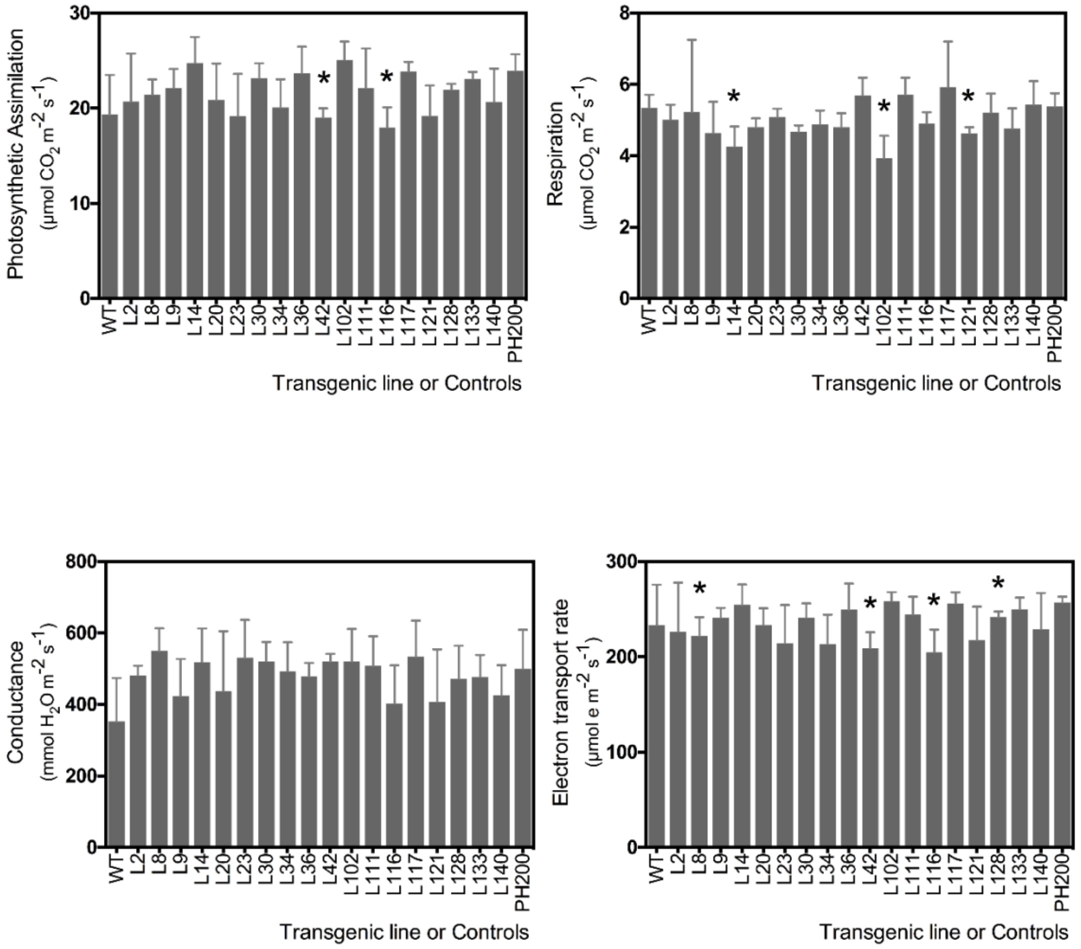

Supplementary Figure S2. Flowering time and days after anthesis (DAP) to get first mature fruit of transgenic lines under glasshouse (A-B) and polytunnel (B-C) conditions. Asterisks indicate values that were determined by Student's t test to be significantly different (*P*<0.05) from the control.

A)

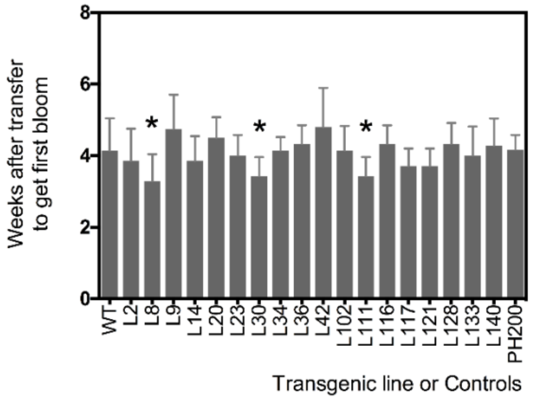

B)

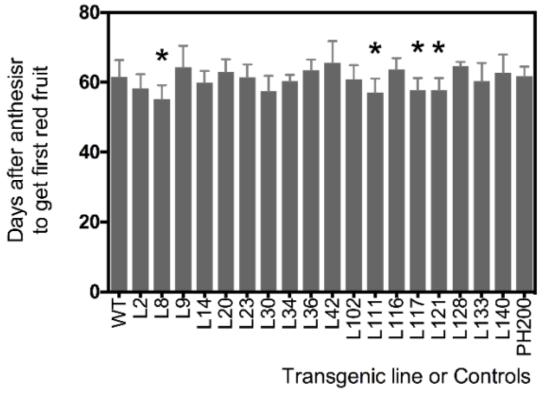

C)

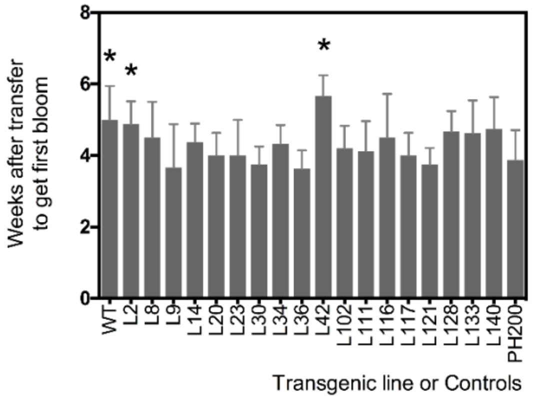

D)

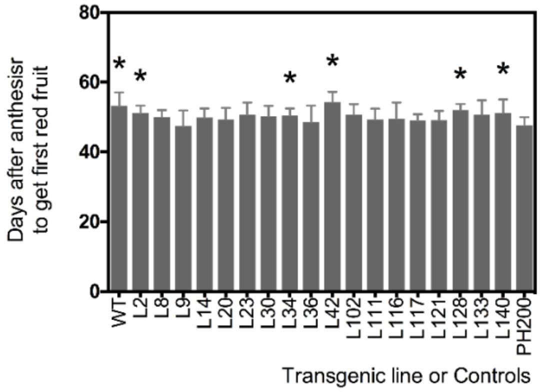

80
60
40
20
0
Days after anthesisr
to get first red fruit
*
*
*
*
*
*
WT
L2
L8
L9
L14
L20
L23
L30
L34
L36
L42
L102
L111
L116
L117
L121
L128
L133
L140
PH200
Transgenic line or Controls

Supplementary Figure S3. Principal Component Analysis (PCA) of the primary metabolite data from selected transgenic lines under glasshouse (A) and polytunnel (B) conditions.

A)

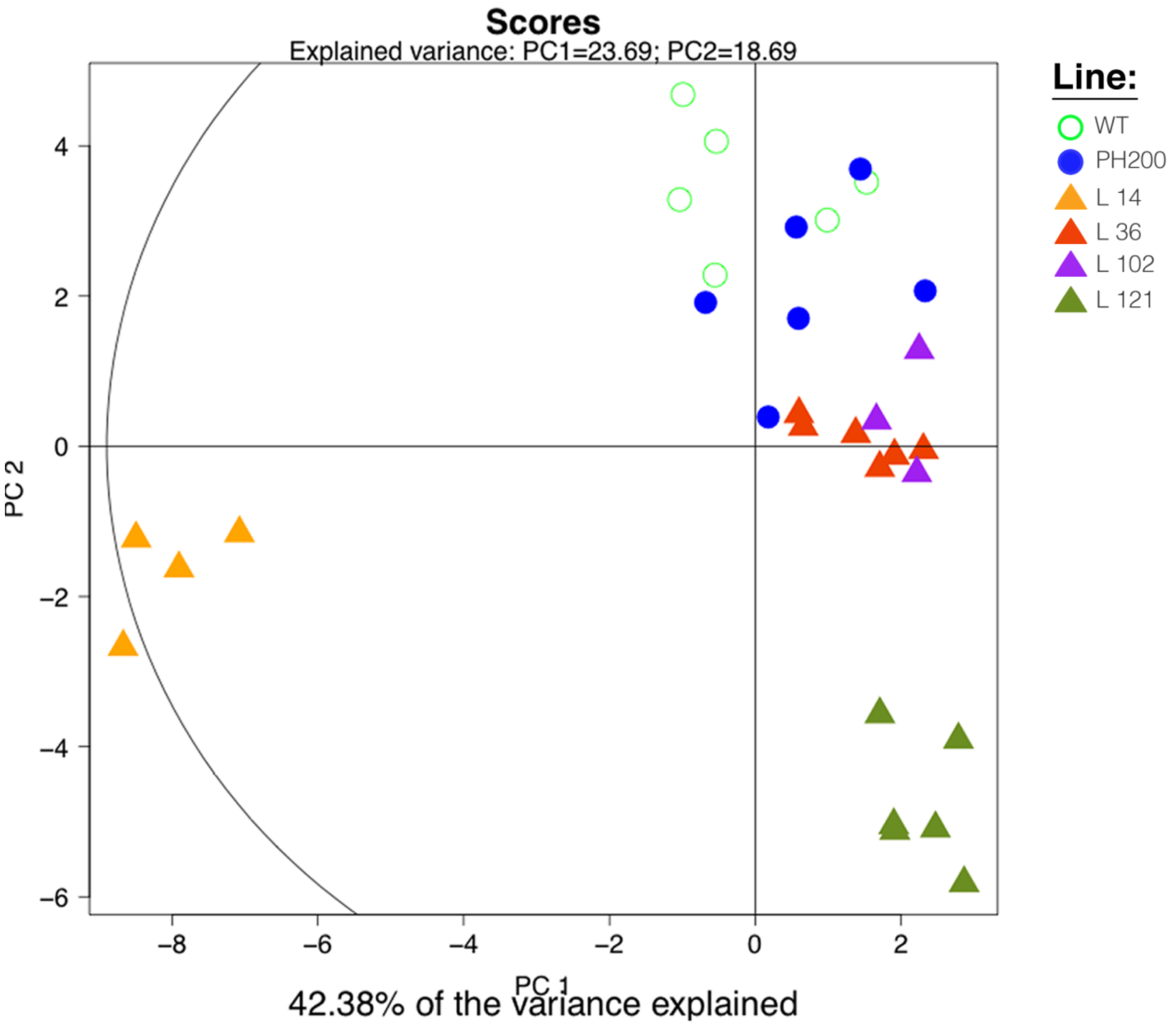

B)

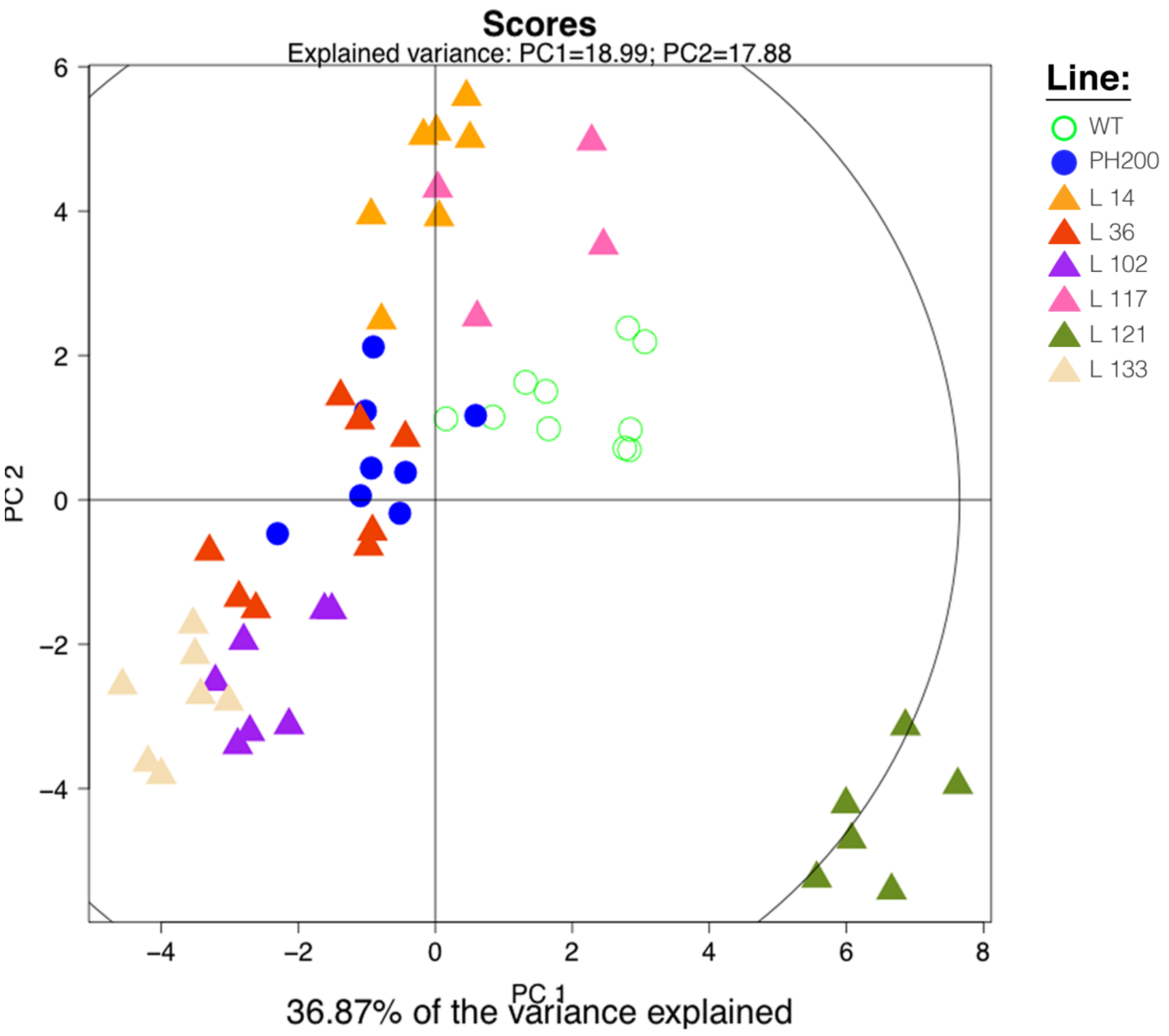

Scores
Explained variance: PC1=18.99; PC2=17.88
PC 2
6
4
2
0
−2
−4
−4
−2
0
2
4
6
8
PC 1
36.87% of the variance explained
Line:
WT
PH200
L 14
L 36
L 102
L 117
L 121
L 133

Supplementary Figure S4. Sparse Partial Least Squares regression (sPLS) model Cor. Circle plot. The variables X and Y are represented through their projections onto the plane defined either by X-variates or Y-variates. The variables X and Y being assumed to be of unit variance, their projections are inside a circle of radius 1 centered at the origin. Strongly associated variables are projected in the same direction from the origin. The greater the distance from the origin the stronger the association. Two circumferences of radius 1 and 0.5 are plotted to reveal the correlation structure of the variables under glasshouse (A) and polytunnel (B) conditions.

A)

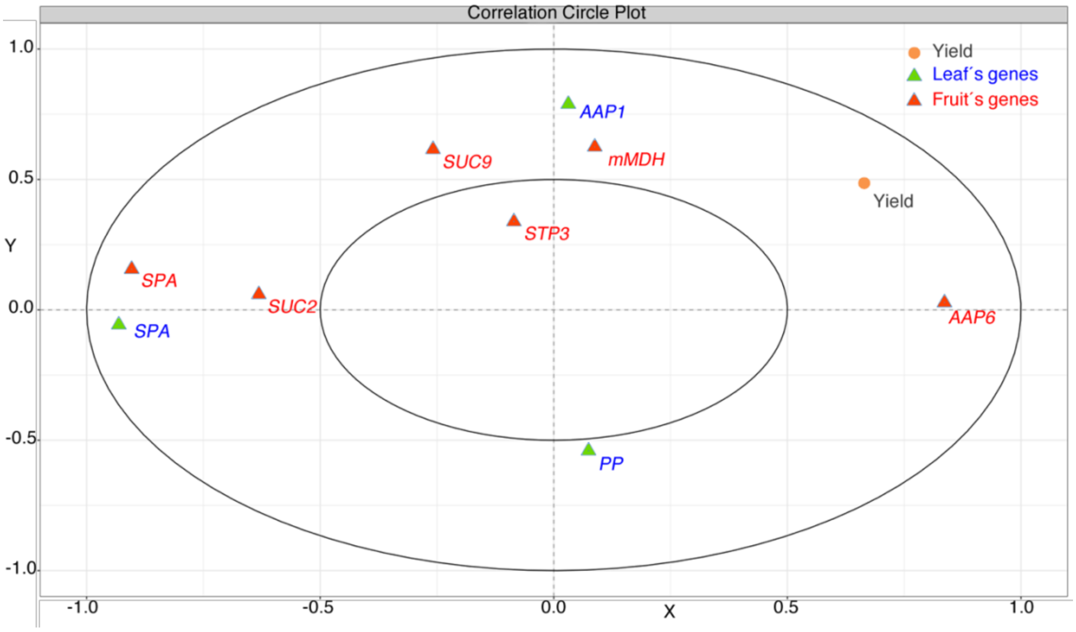

B)

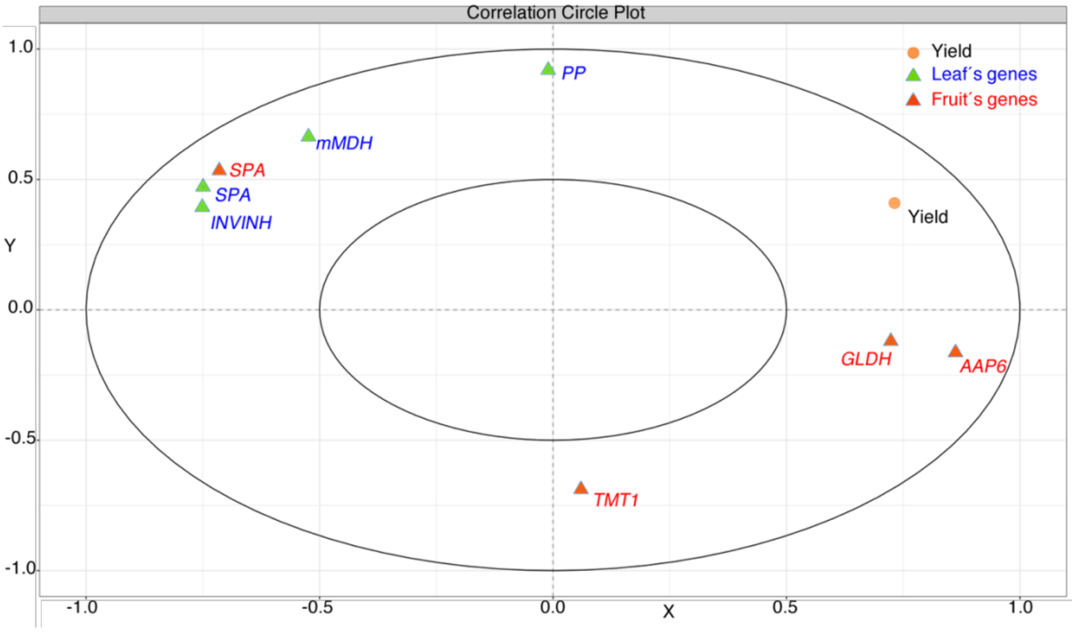
Correlation Circle Plot
Yield
Leaf´s genes
Fruit´s genes
PP
mMDH
SPA
SPA
INVINH
Yield
GLDH
AAP6
TMT1
1.0
0.5
0.0
-0.5
-1.0
Y
-1.0
-0.5
0.0
0.5
1.0
X

Supplementary Note

The selected genes can be classified by three different processes of carbon and nitrogen metabolism; (i) assimilation (*SlmMDH*, *Solanum lycopersicum* mitochondrial malate dehydrogenase[75]; *AtSBP*, *Arabidopsis thaliana* sedoheptulose 1,7-bisphosphatase[98,99]; *SlSPA*, *S. lycopersicum* sugar partitioning affected[68]; *EcPP*, *Escherichia coli pyrophosphatase*[39]; *NtGS2*, *Nicotiana tabacum* chloroplast glutamine synthetase 2[100]; *FpGLDH, Flaveria pringlei* H-protein of glycine decarboxylase[79]; (ii) transport (*AtSWEET11, A. thaliana* sugar efflux transporter 11[101]; *AtSUC2, A. thaliana* sucrose transporter 2[102,103]; *AtAAP1, A. thaliana* amino acid permease 1[104]; and (iii) sink metabolism (*AtSUC2/9, A. thaliana* sucrose transporter 2/9[102,103]; *AtSTP3/6*, *A. thaliana* sugar transporter 3/6[110]; *SpLIN5, S. pennellii* tomato apoplastic invertase 5[80]; *SlINVINH, S. lycopersicum* apoplastic invertase inhibitor[74], *AtSUS1, A. thaliana* sucrose synthase 1[106]; *ShAgpL1, S. habrochaites* Large subunit of ADPglucose pyrophosphorylase 168; *AtTMT1, A. thaliana* tonoplast monosaccharide transporter 1[108]; *AtAAP6, A. thaliana* amino acid permease 6[104]; *SlCAT9, S. lycopersicum* cationic amino acid transporter 9[105]. Overexpression or silencing of these genes were achieved using seven different tissue specific promoters; (i) leaf- and mesophyll-specific: ribulose-bisphosphate carboxylase (RbcS), and cytosolic fructose-1,6-bisphosphate (cyFBP), respectively; (ii) constitutive: cauliflower mosaic virus 35S promoter (35S); (iii) companion cell-specific: commelina yellow mottle virus (CoYMV); (iv) fruit specific: patatin B33 (B33), and ripening-specific ethylene-inducible E8 (E8); and (v) native promoter of *S. habrochaites* Large subunit of ADPglucose pyrophosphorylase 1 (ShAgpL1), which is characterized by increased activity in young fruits[68].

Supplementary Data

Evaluation of this entire gene expression dataset by two-way ANOVA analysis on leaves and fruits separately, revealed that most of genes are influenced significantly by genotype (line) suggesting differential gene expression responses of individual lines to the two growth conditions. When the effect of growth conditions and the interaction of genotype and growth conditions were tested, ANOVA analysis revealed far fewer genes were influenced (Supplementary Table S2). In leaves, only three genes (*AtSUS1*, *AtTMT1*, and *AtSUC9*) on a few transgenic lines were significantly influenced by the interaction of growth conditions and genotype, suggesting that only these three genes responded to the tested growth conditions in a specific manner. On the other hand, ANOVA analysis on fruits, revealed that only *EcPP*, *FpGLDH*, and *AtAAP1* gene expression on few transgenic lines were affected by interaction of growth conditions and genotype (Supplementary Table S2).

Supplementary Discussion

We were not able to find strong and common statistical support for many such features, we made some interesting observations. Sucrose levels increase during tomato fruit ripening. This rise may be due to the incoming sucrose from the

photosynthate translocation from the leaf, where it is loaded into phloem in either an apoplastic or a symplastic manner[80,81,111,112]. This sugar is likely used to support respiration, as well as providing substrate to be metabolized into storage polymers and into primary metabolites needed for growth[113]. We observed a slight increase in sucrose levels correlated with similar increase in fructose, fructose 6-P, in mature fruit irrespective of the growth conditions indicating that the different amount of sugars in fruit may be dependent on endogenous metabolic processes. However, we cannot currently formally discard differences in the degree of phloem unloading, since tomato fruit has been demonstrated to have low photosynthetic activity[114]. This is particularly evident when it is considered that previous studies suggest that sucrose import ceases during tomato fruit ripening due to the formation of an abscission layer between the calyx and fruit[115,116].

In plants grown under high light condition (polytunnel), we observed a general decrease in the levels of raffinose. Unlike the situation observed in the Curcubitaceae, raffinose does not constitute a significant component of phloem-transported sugars in tomato[117]. However, there could conceivably be a role for raffinose, in stress tolerance since it has been implicated to have membrane stabilization and antioxidative functions[118-120]. Raffinose is synthesized by transferring a galactose residue from galactinol to sucrose, and *myo*-inositol is used to synthetize galactinol[121]. In addition, *myo* -inositol itself is implicated to function as an osmolyte to enhance tolerance to abiotic stress[122]. Consistently, galactinol was reduced in these fruits. The lower raffinose level may indicate the use of the raffinose family of oligosaccharides as carbon sources[119].

During normal tomato ripening at the initiation of ethylene biosynthesis, aspartic acid increases in addition to putrescine, one of the three major plant polyamines[123]. Recently, ethylene and polyamines have been reported to possess opposing biological roles: ethylene promotes senescence, whereas polyamines are known to suppress it, by slowing down membrane deterioration and loss of chlorophyll and enhancing protease and RNAse activities[124]. Interestingly, in mature fruits from transgenic plants grown under high light intensity, we observed high increase in aspartate acid level while putrescine decreased in comparison to control plants; however, we observed the opposite behavior in plants grown under low light intensity and limited soil conditions (greenhouse). Further investigations are, however, needed to examine the biological significance of these changes.

It has been long documented that proline accumulates under stress conditions[125,126], as well as being one of many well-known compatible solutes in plants[127]. We observed accumulation of proline in mature fruit in the high yielding transgenic plants grown in high light conditions, indicating it may confer tolerance. Another general trend was the commonly increased level of phenylalanine

observed in mature fruit from the high-yielding transgenic plants grown in the greenhouse while the opposite behavior was observed in fruits from the same plants grown in high light conditions. Interestingly, aromatic amino acids can act as alternative respiratory substrates in instances in which carbohydrates are not abundant[128]. Intriguingly, the mitochondrial electron transfer system which renders this possible is very highly expressed in tomato fruit tissues[129,130], suggesting that this metabolic shift may support the enhanced energy requirements associated with elevate fruit growth in the greenhouse but that another mechanism is invoked under high light conditions when the phenylalanine is likely utilized in the production of phenylpropanoid sunscreen[131].
